# Supplementary figures and images for: A Salmonella type III effector, PipA, works in a different manner than the PipA family effectors GogA and GtgA
Source: PLoS One. 2021 Mar 18;16(3):e0248975. doi: 10.1371/journal.pone.0248975 (PMC7971870; doi:10.1371/journal.pone.0248975)

**A**

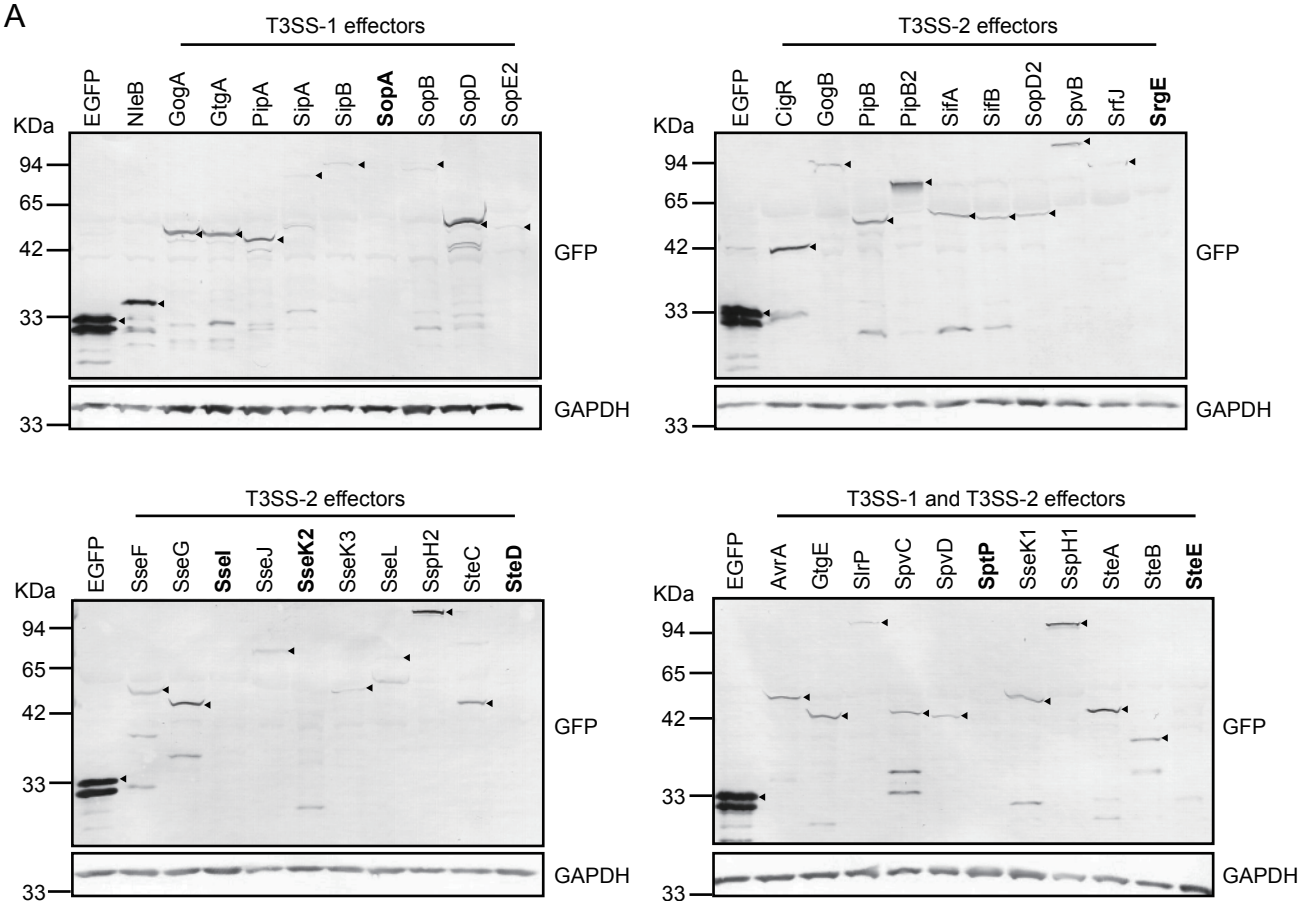

**B**

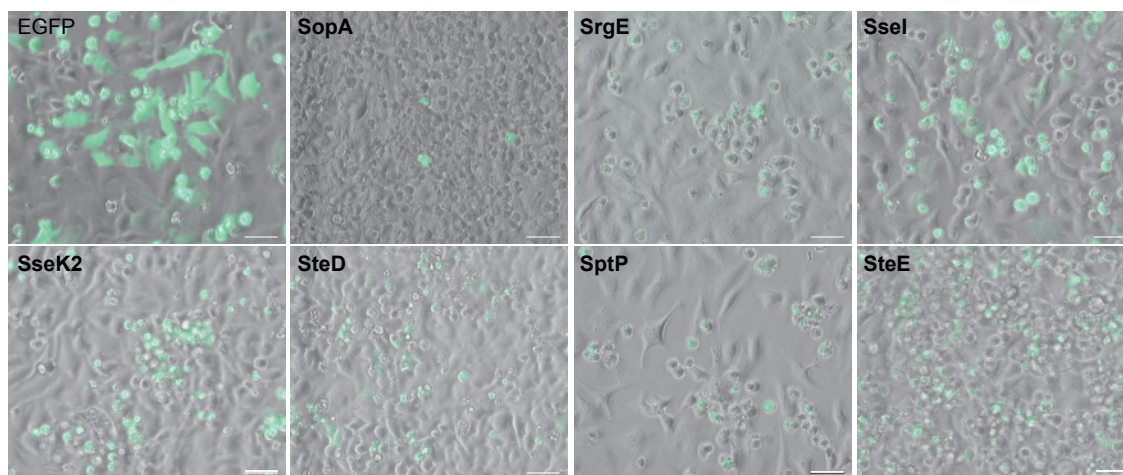

S1 Fig

Supplement: S1 Fig — (A) Representative immunoblots of the EGFP-effector fusion protein in HeLa cells transfected with pEGFP-C1 or pEGFP-effector plasmids. HeLa cells were transfected with pEGFP-C1 or the indicated pEGFP-effector fusion plasmid. After 48 hrs of transfection, cells were lysed with 1 × SDS sample buffer. Total proteins in the whole lysate were separated by SDS-PAGE, and EGFP or the EGFP fusion proteins were detected with immunoblotting using an anti-EGFP antibody. Arrows: The EGFP or EGFP-effector fusion proteins (α-GFP antibody). GAPDH was used as a loading control. (B) Fluorescent microscopy images of HeLa cells transfected with the indicated pEGFP-fusion plasmid (green), which was not detected by immunoblotting (effectors indicated in boldface in the panel A). HeLa cells were transfected with pEGFP-C1 or the indicated pEGFP-effector fusion plasmid. After 48 hrs of transfection, cells were visualized using a fluorescence microscope. (PDF) [file pone.0248975.s001.pdf]

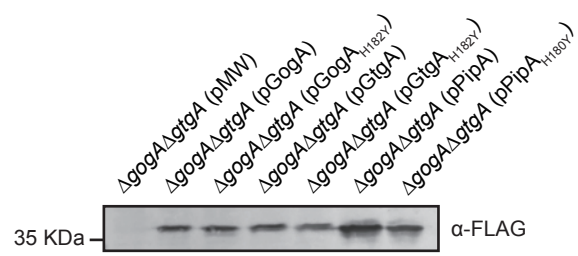

S2 Fig

Supplement: S2 Fig — The indicated bacterial strains were incubated for 15–20 hrs. Overnight bacterial cultures were diluted 1:33 in LB and incubated for 3 hrs in LB containing 0.3M NaCl and 1 μM Isopropyl β-D-1-thiogalactopyranoside (IPTG) to induce expressions of T3SS-1 or the effector gene from a tac promoter on the plasmids, respectively. The FLAG-fusion proteins were detected by immunoblotting using an anti-FLAG antibody. (PDF) [file pone.0248975.s002.pdf]

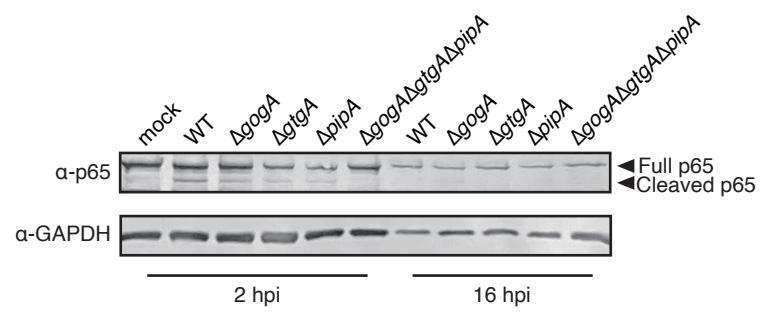

S3 Fig

Supplement: S3 Fig — HeLa cells were infected with the indicated S. Typhimurium strains and the gentamycin killing assay was performed. After 2 or 16 hrs of infection, the cells were detached with TrypLE Select and the cytoplasmic fraction was isolated. The full-length p65 (Full p65) or cleaved p65 in the cytoplasmic fraction was detected by immunoblotting using an anti-p65 antibody. Immunoblots are representative of at least three independent experiments. (PDF) [file pone.0248975.s003.pdf]

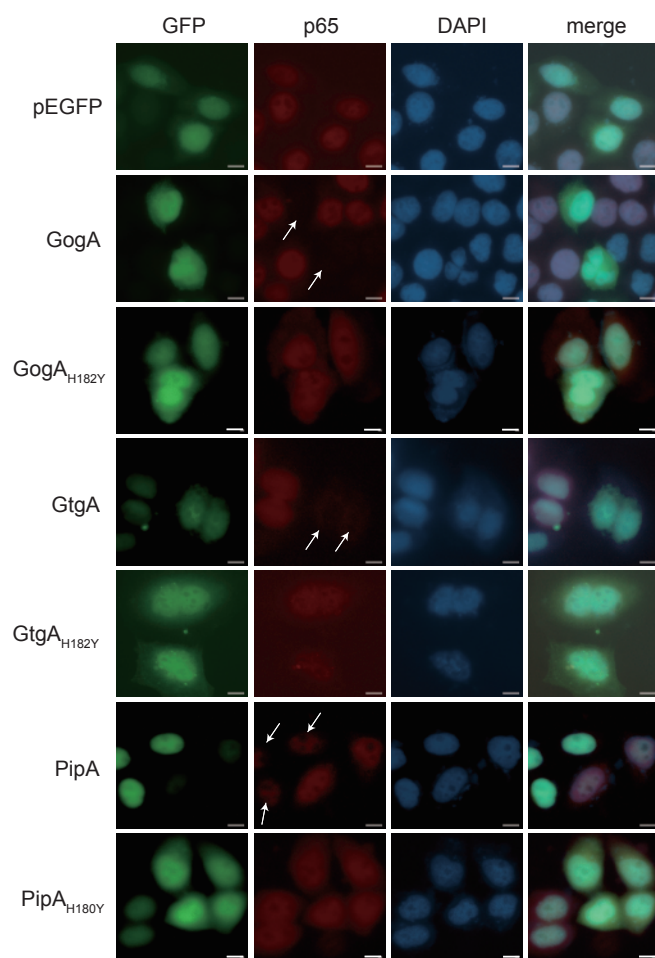

S4 Fig

Supplement: S4 Fig — Fluorescent microscopy images of HeLa cells transfected with the indicated pEGFP-fusion plasmid are shown. HeLa cells were transfected with the indicated pEGFP fusion plasmid (green). After 48 hrs, the cells were stimulated with TNF-α (10 ng/ml) and then further cultured for 30 min and treated with anti-NF-κB subunit p65 antibodies (red) and DAPI (blue) to stain the nuclei. White arrows: The cells inhibiting the translocation of p65 into the nucleus by GogA, GtgA, or PipA. Scale bar, 10 μm. (PDF) [file pone.0248975.s004.pdf]

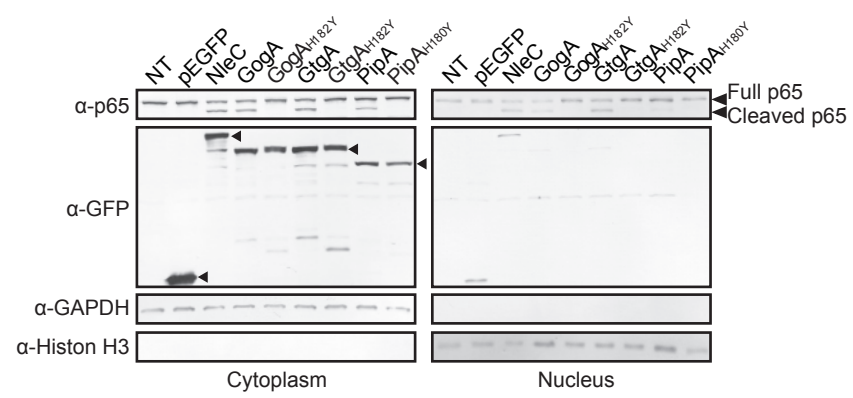

S5 Fig

Supplement: S5 Fig — Representative immunoblots of p65 in HEK293T cells transfected with the pEGFP-C1 or pEGFP effectors from three independent experiments. Arrows: Full p65 or cleaved p65 (α-p65 antibody), or EGFP or EGFP-effector fusion proteins (α-GFP antibody). Histone H3 and GAPDH were used as a loading control in the nucleus and the cytoplasm fraction, respectively. (PDF) [file pone.0248975.s005.pdf]

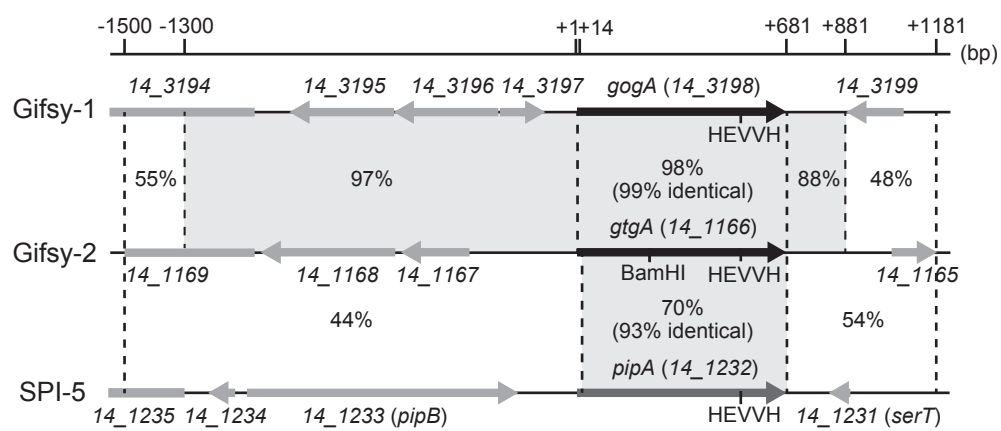

S6 Fig

Supplement: S6 Fig — The initial nucleotide on the gogA, gtgA, or pipA gene is indicated as +1. Arrows and the numbers above/beyond the arrows show the gene coding sequence (CDS) and the CDS names annotated for the genome of S. Typhimurium ATCC 14028, respectively. The % indicates the homology of the DNA sequence enclosed by dotted lines. (PDF) [file pone.0248975.s006.pdf]

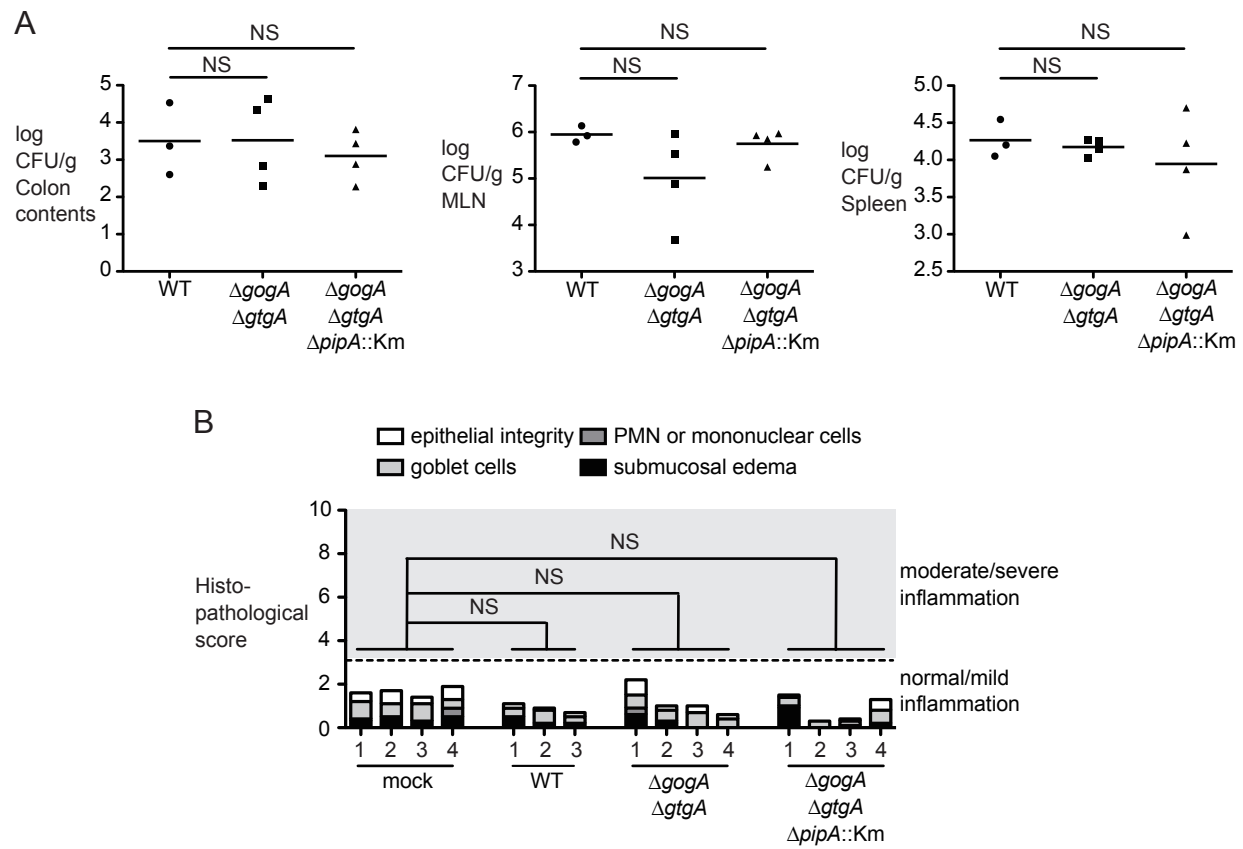

S7 Fig

Supplement: S7 Fig — CBA mice were infected intragastrically with 1×10^9^ CFU of the indicated S. Typhimurium strains. (A) Bacterial numbers recovered from the colon contents, mesenteric lymph nodes (MLNs), and spleen at 4 days after infection. Individual data are shown as a scatter plot, and bars are the mean. (B) Histopathological changes were scored in sections of the cecum at 4 days after infection. Each bar represents the combined scoring results for a single mouse. (PDF) [file pone.0248975.s007.pdf]

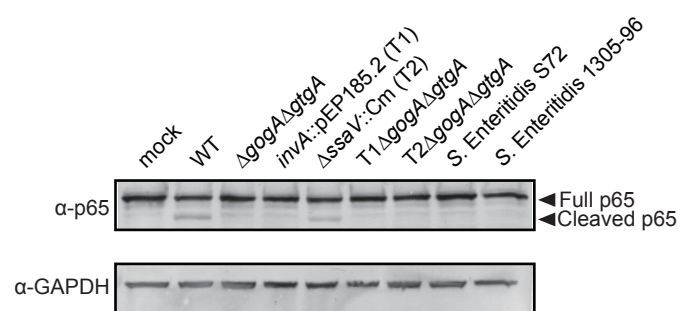

S8 Fig

Supplement: S8 Fig — The cleavage of p65 in HeLa cells infected with the indicated S. Typhimurium or S. Enteritidis strain was detected by immunoblotting using an anti-p65 antibody. (PDF) [file pone.0248975.s008.pdf]

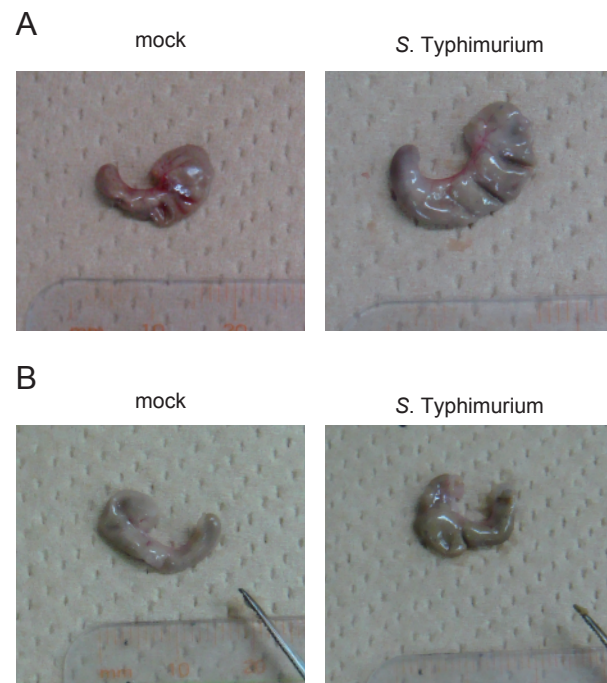

S9 Fig

Supplement: S9 Fig — (A) Cecum of CBA mice infected with the S. Typhimurium wild-type strain or the ΔgogAΔgtgAΔpipA mutant at 13 days after infection. (B) Cecum of CBA mice from Charles River Japan infected with the S. Typhimurium wild-type strain or the ΔgogAΔgtgAΔpipA mutant at 4 days after infection. (PDF) [file pone.0248975.s009.pdf]
