# Supplementary material for: A Salmonella type III effector, PipA, works in a different manner than the PipA family effectors GogA and GtgA
Source: PLoS One. 2021 Mar 18;16(3):e0248975. doi: 10.1371/journal.pone.0248975 (PMC7971870; doi:10.1371/journal.pone.0248975)
Supplement: S1 Table — (PDF) [file pone.0248975.s010.pdf]

S1 Table. Bacterial strains and plasmids used in this study

Strains	Relevant characteristics	Source/Ref.
S. enterica serovar Typhimurium		
SH100	Nalidixic acid-resistant derivative of wild-type 14028 (WT)	[1]
TH1671	SH100 Δ*gogA*::Cm	this study
TH1678	SH100 Δ*gtgA*::Cm	this study
TH1772	SH100 Δ*pipA*::Km	this study
TH1681	SH100 Δ*gogA* Δ*gtgA*	this study
TH1821	SH100 Δ*gogA* Δ*pipA*::Km	this study
TH1820	SH100 Δ*gtgA* Δ*pipA*::Km	this study
TH1779	SH100 Δ*gogA* Δ*gtgA* Δ*pipA*::Km	this study
TH1624	SH100 *invA*::pEP185.2 (T1)	this study
SH113	SH100 Δ*ssaV*::Cm (T2)	[2]
TH1722	SH100 Δ*gogA* Δ*gtgA* Δ*pipA* Δ*sseK1* Δ*sseK2* Δ*sseK3*::Km Δ*steE*::Cm	this study
TH1586	SH100 Δ*sseK1* Δ*sseK2* Δ*sseK3*::Km	this study
TH1761	SH100 Δ*steE*::Cm	this study
TH2133	SH100 *invA*::pEP185.2 Δ*pipA*::Km	this study
TH2213	SH100 *invA*::pEP185.2 Δ*pipA*::Km *phoN*::*pipA*	this study
TH2214	SH100 *invA*::pEP185.2 Δ*pipA*::Km *phoN*::$pipA_{H180Y}$	this study
TH1766	SH100 *invA*::pEP185.2 Δ*gogA* Δ*gtgA*	this study
TH1770	SH100 Δ*ssaV*::Cm Δ*gogA* Δ*gtgA*	this study
S. enterica serovar Enteritidis		
S72	clinical isolate	National Institute of Infectious Diseases
1305-96	clinical isolate	National Institute of Infectious Diseases
E. coli		
DH5α	K-12 *recA1 endA1 gyrA96 thi-1 hsdR17 supE44* (*lacXYA-argR*) *U169 deoR* (*80 dlac* (*lacZ*) *M15*)	Invitrogen
BL21(DE3)	F- *ompT hsdSB* (rB- mB-) *gal dcm* (DE3)	NEB
