# Supplementary material for: A Salmonella type III effector, PipA, works in a different manner than the PipA family effectors GogA and GtgA
Source: PLoS One. 2021 Mar 18;16(3):e0248975. doi: 10.1371/journal.pone.0248975 (PMC7971870; doi:10.1371/journal.pone.0248975)
Supplement: S2 Table — (PDF) [file pone.0248975.s011.pdf]

S2 Table. Plasmids used in this study

Plasmid	Relevant characteristics	Source/Ref.
pTAKN-2	TA-cloning vector, Km^R	BioDynamics Laboratory
pMW118 (pMW)	pSC101-based low copy number plasmid, Ap^R	Nippon Gene
pGogA	*gogA* cloned on pMW118	this study
pGogA$_{H182Y}$	*gogA* H182Y cloned on pMW118	this study
pGtgA	*gtgA* cloned on pMW118	this study
pGtgA$_{H182Y}$	*gtgA* H182Y cloned on pMW118	this study
pPipA	pipA cloned in pMW118	this study
pEGFP-C1	N-terminal EGFP mammalian expression vector, Km^R	Clonetech
pEGFP-NleB	EPEC *nleB1* cloned on pEGFP-C1	this study
pEGFP-NleC	EPEC *nleC* cloned on pEGFP-C1	[3]
pEGFP-SpvC	*spvC* cloned on pEGFP-C1	[4]
pEGFP-GogA	*gogA* cloned on pEGFP-C1	this study
pEGFP-GogA$_{H182Y}$	*gogA*$_{H182Y}$ cloned on pEGFP-C1	this study
pEGFP-GtgA	*gtgA* cloned on pEGFP-C1	this study
pEGFP-GtgA$_{H182Y}$	*gtgA*$_{H182Y}$ cloned on pEGFP-C1	this study
pEGFP-PipA	*pipA* cloned on pEGFP-C1	this study
pEGFP-PipA$_{H180Y}$	*pipA*$_{H180Y}$ cloned on pEGFP-C1	this study
pEGFP-SipA	*sipA* cloned on pEGFP-C1	this study
pEGFP-SipB	*sipB* cloned on pEGFP-C1	this study
pEGFP-SopA	*sopA* cloned on pEGFP-C1	this study
pEGFP-SopB	*sopB* cloned on pEGFP-C1	this study
pEGFP-SopD	*sopD* cloned on pEGFP-C1	this study
pEGFP-SopE2	*sopE2* cloned on pEGFP-C1	this study
pEGFP-CigR	*cigR* cloned on pEGFP-C1	this study
pEGFP-GogB	*gogB* cloned on pEGFP-C1	this study
pEGFP-PipB	*pipB* cloned on pEGFP-C1	this study
pEGFP-PipB2	*pipB2* cloned on pEGFP-C1	this study
pEGFP-SifA	*sifA* cloned on pEGFP-C1	this study

pEGFP-SifB	*sifB* cloned on pEGFP-C1	this study
pEGFP-SopD2	*sopD2* cloned on pEGFP-C1	this study
pEGFP-SpvB	*spvB* cloned on pEGFP-C1	this study
pEGFP-SrfJ	*srfJ* cloned on pEGFP-C1	this study
pEGFP-SrgE	*srgE* cloned on pEGFP-C1	this study
pEGFP-SseF	*sseF* cloned on pEGFP-C1	this study
pEGFP-SseG	*sseG* cloned on pEGFP-C1	this study
pEGFP-SseI	*sseI* cloned on pEGFP-C1	this study
pEGFP-SseJ	*sseJ* cloned on pEGFP-C1	this study
pEGFP-SseK2	*sseK2* cloned on pEGFP-C1	this study
pEGFP-SseK3	*sseK3* cloned on pEGFP-C1	this study
pEGFP-SseL	*sseL* cloned on pEGFP-C1	this study
pEGFP-SspH2	*sspH2* cloned on pEGFP-C1	this study
pEGFP-SteC	*steC* cloned on pEGFP-C1	this study
pEGFP-SteD	*steD* cloned on pEGFP-C1	this study
pEGFP-AvrA	*avrA* cloned on pEGFP-C1	this study
pEGFP-GtgE	*gtgE* cloned on pEGFP-C1	this study
pEGFP-SlrP	*slrP* cloned on pEGFP-C1	this study
pEGFP-SpvD	*spvD* cloned on pEGFP-C1	this study
pEGFP-SptP	*sptP* cloned on pEGFP-C1	this study
pEGFP-SseK1	*sseK1* cloned on pEGFP-C1	this study
pEGFP-SspH1	*sspH1* cloned on pEGFP-C1	this study
pEGFP-SteA	*steA* cloned on pEGFP-C1	this study
pEGFP-SteB	*steB* cloned on pEGFP-C1	this study
pEGFP-SteE	*steE* cloned on pEGFP-C1	this study
pGEX-6P-1	N-terminal GST expression vector, Ap^R	GE Healthcare
pGEX-NleC	*nleC* cloned on pGEX-6P-1	this study
pGEX-GogA	*gogA* cloned on pGEX-6P-1	this study
pGEX-$GogA_{H182Y}$	$gogA_{H182Y}$ cloned on pGEX-6P-1	this study
pGEX-GtgA	*gtgA* cloned on pGEX-6P-1	this study
pGEX-$GtgA_{H182Y}$	$gtgA_{H182Y}$ cloned on pGEX-6P-1	this study

pGEX-PipA	*pipA* cloned on pGEX-6P-1	this study
pGEX-PipA_{H180Y}	*pipA*$_{H180Y}$ cloned on pGEX-6P-1	this study
pFLAG-CTC	FLAG fusion vector, Ap^R	Sigma
pFLAG-GogA	*gogA* cloned on pFLAG-CTC	this study
pFLAG-GogA_{H182Y}	*gogA*$_{H182Y}$ cloned on pFLAG-CTC	this study
pFLAG-GtgA	*gtgA* cloned on pFLAG-CTC	this study
pFLAG-GtgA_{H182Y}	*gtgA*$_{H182Y}$ cloned on pFLAG-CTC	this study
pFLAG-PipA	*pipA* cloned on pFLAG-CTC	this study
pFLAG-PipA_{H180Y}	*pipA*$_{H180Y}$ cloned on pFLAG-CTC	this study
pCMV-FLAG-p65	FLAG-p65 expression plasmid	[5]
pSW85	*phoN* franking regions cloned on pGP704	[6]
pSW85Ω	Ω fragment cloned on pSW85	this study
pSW85Ω-pipA	*pipA* cloned on pSW85Ω	this study
pSW85Ω-pipA_{H180Y}	*pipA*$_{H180Y}$ cloned on pSW85Ω	this study
pKD46	Red recombinase expression plasmid, Ap^R	[7]
pCP20	FLP recombinase expression plasmid, Ap^R	[7]
