# Supplementary material for: A Salmonella type III effector, PipA, works in a different manner than the PipA family effectors GogA and GtgA
Source: PLoS One. 2021 Mar 18;16(3):e0248975. doi: 10.1371/journal.pone.0248975 (PMC7971870; doi:10.1371/journal.pone.0248975)
Supplement: S3 Table — (PDF) [file pone.0248975.s012.pdf]

S3 Table. Nucleotide primers used in this study

Purpose	No.	Name	Nucleotide sequence (5' to 3')
Construction of Δ*gogA*::Cm or Δ*gtgA*::Cm	TH788	GogA-KO-F1	AATGTTAATTCCATGTAATAAAAAGGATGTGTAACTCATCGTGTAGGCTGGAGCTGCTTC
	TH789	GogA-KO-R1	GTGTTGTAGCATCGTGGGATTTTGCATTTTTTGATGAGTGCATATGAATATCCTCCTTAG
Construction of Δ*pipA*::Km	TH827	PipA-KO-F2	CCTTGCCATAAACAGTGAATTCCGTCTGGAGCCAGAGTGTGTGTAGGCTGGAGCTGCTTC
	TH828	PipA-KO-R2	ACTACGCGAGTCTTTAGTTTCTTTTCGTTTCCCGATGTGTCATATGAATATCCTCCTTAG
Construction of Δ*sseK1*::Km	NO01	SseK1-KO-F1	TAAAATATGTAATGAAGTAAGTATGGAGCATTTAATTGTTGTGTAGGCTGGAGCTGCTTC
	NO02	SseK1-KO-R1	GCCTCGCCCATGAACTTTGCGTAAACTGACTGGTATTCATCATATGAATATCCTCCTTAG
Construction of Δ*sseK2*::Km	NO03	SseK2-KO-F1	AAATCATGGTATGTTATATTAATAGCGTAAGGGTTGAAAAGTGTAGGCTGGAGCTGCTTC
	NO04	SseK2-KO-R1	CGAAACATTGCTCGCGTTTATATCATACGTTTGCAAATAACATATGAATATCCTCCTTAG
Construction of Δ*sseK3*::Km	TH367	SseK3-KO-F1	AAATCATGGTATGTTATATTAATAGCGTAAGGGTTGAAAAGTGTAGGCTGGAGCTGCTTC
	TH368	SseK3-KO-R1	CGAAACATTGCTCGCGTTTATATCATACGTTTGCAAATAACATATGAATATCCTCCTTAG
Construction of Δ*steE*::Cm	TH798	SteE-KO-F2	TCTTTAAAAACTACTGCATGTAAAAGGGTCTCCTCTTGTTGTGTAGGCTGGAGCTGCTTC

	TH799	SteE-KO-R2	GTGATCTACTATTCGGCGCAGCTATTTATAACGCTTTGTTCATATGAATATCCTCCTTAG
Construction of pGogA or pGtgA	TH782	GtgA-F4-SacI	GGGGAGCTCAAGCCTCACCTCCGATGATTTCGGA
	TH783	GtgA-R4-SphI	GGGGCATGCGACAGGAGCCAGTGTCTATGGACTG
Construction of pPipA	TH830	PipA-F3-SacI	GGGGAGCTCTCGAGATAAAGGGATTTTATAAACA
	TH831	PipA-R3-EcoR!	GGGGCATGCGATTAAACAAGGGGTTACCGAAAGG
Construction of $GogA_{H182Y}$	TH816	GogA-SDM-F1	TTTGATACAACACGCTCATTTATTTATGAAGTAGTACACGCGTTG
	TH817	GogA-SDM-R1	CAACGCGTGTACTACTTCATAAATAAATGAGCGTGTTGTATCAAA
Construction of $GtgA_{H182Y}$	TH765	GtgA-SDM-F1	TTTGATACAACACGCTCATTTATTTATGAAGTTGTACACGCGTTG
	TH766	GtgA-SDM-R1	CAACGCGTGTACAACTTCATAAATAAATGAGCGTGTTGTATCAAA
Construction of $PipA_{H180Y}$	TH846	GogA-SDM-F1	GACAACTTTTTGACACAAAACGTTCATTTATTTATGAAGTTGTACATGCC
	TH847	GogA-SDM-R1	GGCATGTACAACTTCATAAATAAATGAACGTTTTGTGTCAAAAAGTTGTC
Construction of pEGFP-GogA or pEGFP-GtgA	TH683	GogA-F1-XhoI	GGCTCGAGTGCCAACGGGAATTAAACCAATATT
	TH684	GogA-R1-BglII	GGAGATCTTCAATTACTAAATTCGTAGGCGATT
Construction of pEGFP-PipA	TH774	PipA-F1-XhoI	GGGCTCGAGTGGTGGAGTACCTTATCTCAGGCGC
	TH775	PipA-R1-BamHI	GGGGATCCCTATTTATTGAAGATGTAGACCATT
Construction of pEGFP-SipA	TH715	SipA-F2-BamHI	GGGGATCCGTTACAAGTGTAAGGACTCAGCCCC
	TH716	SipA-R2-XhoI	GGCTCGAGTTAACGCTGCATGTGCAAGCCATCA
Construction of pEGFP-SipB	TH646	SipB-R1-BamHI	GGGGATCCTTATGCGCGACTCTGGCGCAGAATA

	TH647	SipB-F1-XhoI	GGCTCGAGTGGTAAATGACGCAAGTAGCATTAG
Construction of pEGFP-SopA	TH719	SopA-F2-BamHI	GGGGATCCAAGATATCATCAGGCGCAATTAATT
	TH720	SopA-R2-XhoI	GGCTCGAGCTACGCCCAGGCCAGTGGCAGGATG
Construction of pEGFP-SopB	TH721	SopB-F2-BamHI	GGGGATCCCAAATACAGAGCTTCTATCACTCAG
	TH722	SopB-R2-XhoI	GGCTCGAGTCAAGATGTGATTAATGAAGAAATG
Construction of pEGFP-SopD	TH654	SopD-R1-XhoI	GGCTCGAGTGCCAGTCACTTTAAGCTTCGGTAA
	TH655	SopD-F1-BamHI	GGGGATCCTTATGTCAGTAATATATTACGACTG
Construction of pEGFP-SopE2	TH656	SopE2-R1-BamHI	GGGGATCCTCAGGAGGCATTCTGAAGATACTTA
	TH657	SopE2-F1-XhoI	GGCTCGAGTGACTAACATAACACTATCCACCCA
Construction of pEGFP-CigR	TH687	CigR-F1-XhoI	GGCTCGAGTGAATAATCGTCGTGGTTTAACCGC
	TH688	CigR-R1-BamHI	GGGGATCCTTAATCAAATACGCCATTAATAATC
Construction of pEGFP-GogB	TH660	GogB-R1-XhoI	GGGGATCCTCAACGATTTCTATTTTTAGGCTTA
	TH661	GogB-F1-BamHI	GGCTCGAGTGACATATAGATTGAAAAAGCGCAT
Construction of pEGFP-PipB	TH707	PipB-F2-XhoI	GGCTCGAGTGCCAATAACTAACGCGTCCCCAGA
	TH708	PipB-R2-EcoRI	GGAATTCCTAAAATATCGGATGGGGGAAAAGA
Construction of pEGFP-PipB2	TH669	PipB2-R2-XhoI	GGGGATCCCTAAATATTTTCACTATAAAATTCG
	TH670	PipB2-F2-BamHI	GGCTCGAGTGGAGCGTTCACTCGATAGTCTGGC
Construction of pEGFP-SifA	TH709	SifA-F2-XhoI	GGCTCGAGTGCCGATTACTATAGGGAATGGTTT

	TH710	SifA-R2-EcoRI	GGGAATTCTTATAAAAAACAACATAAACAGCCG
Construction of pEGFP-SifB	TH679	SifB-F1-XhoI	GGCTCGAGTGCCAATTACTATCGGGAGAGGATT
	TH680	SifB-R1-BamHI	GGGGATCCTCAACTCTGGTGATGAGCCTCATTT
Construction of pEGFP-SopD2	TH623	SopD2-F1-XhoI	GGGCTCGAGTGCCAGTTACGTTAAGTTTTGGTAA
	TH624	SopD2-R1-BamHI	GGGGGATCCTTATATAAGCATATTGCGACAACTC
Construction of pEGFP-SpvB	TH1077	SpvB-FW-XhoI	GGGCTCGAGTGTTGATACTAAATGGTTTTTCATC
	TH1078	SpvB-RV-SalI	GGGGTCGACCTATGAGTTGAGTACCCTCATGTTT
Construction of pEGFP-SrfJ	TH685	SrfJ-F1-XhoI	GGCTCGAGTGAAAGGCAGACTCATCTCTTCCGA
	TH686	SrfJ-R1-BamHI	GGGGATCCTCAGATCGACTCCTGCCGCCATAGC
Construction of pEGFP-SrgE	TH825	SrgE-F1-XhoI	GGGCTCGAGTGATGAGTAGTATTACAAAAAGCA
	TH826	SrgE-R1-BamHI	GGGGGATCCCTATTTCTTTTTATATGCCCCATAC
Construction of pEGFP-SseF	TH666	SseF-R1-BamHI	GGGGATCCTTCATGGTTCTCCCCGAGATGTATGA
	TH667	SseF-F1-XhoI	GGCTCGAGTGAAAATTCATATTCCGTCAGCGGC
Construction of pEGFP-SseG	TH662	SseG-R1-BamHI	GGGGATCCTTACTCCGGCGCACGTTGTTCTGGC
	TH663	SseG-F1-XhoI	GGCTCGAGTGAAACCTGTTAGCCCAAATGCTCA
Construction of pEGFP-SseI	TH673	SseI-F1-XhoI	GGCTCGAGTGCCCTTTCATATTGGAAGCGGATG
	TH674	SseI-R1-BamHI	GGGGATCCTTACATTTTACCTATTAAGGAATAT
Construction of pEGFP-SseJ	TH627	SseJ-F1-XhoI	GGCTCGAGTGCCATTGAGTGTTGGACAGGGTTA

	TH631	SseJ-R1-BamHI	GGGGGATCCTTATTCAGTGGAATAATGATGAGCT
Construction of pEGFP-SseK2	TH446	SseK2-F1-BglII	GTCCGGACTCAGATCTATGGCACGTTTTAATGCCGCTTTTA
	TH447	SseK2-R1-BamHI	TAGATCCGGTGGATCCTTACCTCCAAGAACTGGCAGTTAAA
Construction of pEGFP-SseK3	TH448	SseK3-F1-BglII	GTCCGGACTCAGATCTATGTTTTCTCGAGTCAGAGGTTTTC
	TH449	SseK3-R1-BamHI	TAGATCCGGTGGATCCTTATCTCCAGGAGCTGATAGTCAAA
Construction of pEGFP-SseL	TH675	SseL-F1-XhoI	GGCTCGAGTGAATATATGTGTAAATTCACTTTA
	TH676	SseL-R1-BamHI	GGGGATCCTTACTGGAGACTGTATTCATATATT
Construction of pEGFP-SspH2	TH681	SspH2-F1-XhoI	GGCTCGAGTGCCCTTTCATATTGGAAGCGGATG
	TH682	SspH2-R1-BamHI	GGGGATCCTCAGTTACGACGCCACTGAACGTTC
Construction of pEGFP-SteC	TH695	SteC-F1-XhoI	GGCTCGAGTGCCGTTTACATTTCAGATCGGAAA
	TH696	SteC-R1-BamHI	GGGGATCCCTATTTTTTTAATTCATCCTTTAAT
Construction of pEGFP-SteD	TH697	SteD-F1-XhoI	GGCTCGAGTGAACAAATACAGCTATTGCGCAAC
	TH698	SteD-R1-BamHI	GGGGATCCTCAGAAGAAGAAGGTGATGTCTCCG
Construction of pEGFP-AvrA	TH436	AvrA-F1-BglII	GTCCGGACTCAGATCTATGATATTTTCGGTGCAGGAGCTAT
	TH437	AvrA-R1-BamHI	TAGATCCGGTGGATCCTTACGGTTTAAGTAAAGACTTATAT
Construction of pEGFP-GtgE	TH691	GtgE-F1-XhoI	GGCTCGAGTGTTAAGACACATTCAAAATAGTTT
	TH692	GtgE-R1-BamHI	GGGGATCCTCATAAAATGGTACACCAGTCTTTC
Construction of pEGFP-SlrP	TH629	SlrP-F2-HindIII	GGGAAGCTTTGTTTAATATTACTAATATACAATCTACGGCAAGG

	TH630	SlrP-R2-BamHI	GGGGGATCCCTATCGCCAGTAGGCGCTCATGAGCGAGCTCACCT
Construction of pEGFP-SpvD	TH432	SpvD-F1-BglII	GTCCGGACTCAGATCTATGAGAGTTTCTGGTAGTGCGTCAT
	TH433	SpvD-R1-BamHI	TAGATCCGGTGGATCCTCAATCGTGTTTTTCATCATAAGCC
Construction of pEGFP-SptP	TH658	SptP-R2-BamHI	GGGGATCCTCAGCTTGCCGTCGTCGTCATAAGC
	TH659	SptP-F1-XhoI	GGCTCGAGTGCTAAAGTATGAGGAGAGAAAATT
Construction of pEGFP-SseK1	TH444	SseK1-F1-BglII	GTCCGGACTCAGATCTATGATCCCACCATTAAATAGATATG
	TH445	SseK1-C-BamHI	TAGATCCGGTGGATCCCTACTGCACATGCCTCGCCCATGAA
Construction of pEGFP-SspH1	TH423	SspH1-F1-BglII	GTCCGGACTCAGATCTATGTTTAATATCCGCAATACACAAC
	TH429	SspH1-R1-BamHI	TAGATCCGGTGGATCCTCAGTTAAGACGCCACCGGGCTGTC
Construction of pEGFP-SteA	TH693	SteA-F1-XhoI	GGCTCGAGTGCCATATACATCAGTTTCTACCTA
	TH694	SteA-R1-BamHI	GGGGATCCTTAATAATTGTCCAAATAGTTATGG
Construction of pEGFP-SteB	TH611	SteB-F1-XhoI	GGGCTCGAGTGCCTATTTCGATTTGTAAACATGG
	TH612	SteB-R1-BamHI	GGGGGATCCTTATCTGACATTACCATTTGAGTGA
Construction of pEGFP-SteE	TH468	SteE-IF-F1	GTCCGGACTCAGATCTATGAGATTCGTATATATTTATATCT
	TH469	SteE-IF-R1	GCAGAATTCGAAGCTTTTATTCATCCGGGAAAACCTCTGCA
Construction of pEGFP-NleB	TH438	NleB-F1-BglII	GTCCGGACTCAGATCTATGTTATCTTCATTAAATGTCCTTC
	TH439	NleB-R1-BamHI	TAGATCCGGTGGATCCTTACCATGAACTGCTGGTATACATA
	TH778	GtgA-F3-EcoRI	GGGGAATTCCCAGCAGGAATTAAACCAATATTTA

Construction of pGEX-GogA or pGEX-GtgA	TH779	GtgA-R3-XhoI	GGGCTCGAGTCAATTACTAGATTCGTAGGCGATT
Construction of pGEX-PipA	TH831	PipA-F4-EcoRI	GGGGAATTCGTGGTGGAGTACCTTATCTCAGGCG
	TH832	PipA-R4-XhoI	GGGCTCGAGCTATTTATTGAAGATGTAGACCATT
Confirmation of *Mip2* expression (qPCR)	TH46	Mip2-FW	AGTGAACTGCGCTGTCAATGC
	TH47	Mip2-RV	AGGCAAACTTTTTGACCGCC
Confirmation of *Ifng* expression (qPCR)	TH91	IFNg-FW	TCAAGTGGCATAGATGTGGAAGAA
	TH92	IFNg-RV	TGGCTCTGCAGGATTTTCATG
Confirmation of *Tnfa* expression (qPCR)	TH96	TNFa-FW	CATCTTCTCAAAATTCGAGTGACAA
	TH97	TNFa-RV	TGGGAGTAGACAAGGTACAACCC
Reference gene (qPCR)	TH42	GAPDH-FW	TGTAGACCATGTAGTTGAGGTCA
	TH43	GAPDH-RV	AGGTCGGTGTGAACGGATTTG

References

1. Gotoh H, Okada N, Kim Y-G, Shiraishi K, Hirami N, Haneda T, et al. Extracellular secretion of the virulence plasmid-encoded ADP-ribosyltransferase SpvB in *Salmonella*. Microb Pathog. 2003;34: 227–238.

2. Okada N, Oi Y, Takeda-Shitaka M, Kanou K, Umeyama H, Haneda T, et al. Identification of amino acid residues of *Salmonella* SlyA that are critical for transcriptional regulation. Microbiology (Reading, Engl). 2007;153: 548–560. doi:10.1099/mic.0.29259-0

3. Yen H, Ooka T, Iguchi A, Hayashi T, Sugimoto N, Tobe T. NleC, a Type III Secretion Protease, Compromises NF-κB Activation by Targeting p65/RelA. PLoS Pathog. 2010;6: e1001231. doi:10.1371/journal.ppat.1001231.s012

4. Haneda T, Ishii Y, Shimizu H, Ohshima K, Iida N, Danbara H, et al. *Salmonella* type III effector SpvC, a phosphothreonine lyase, contributes to reduction in inflammatory

response during intestinal phase of infection. Cell Microbiol. 2012;14: 485–499. doi:10.1111/j.1462-5822.2011.01733.x

5. Ashida H, Nakano H, Sasakawa C. *Shigella* IpaH0722 E3 Ubiquitin Ligase Effector Targets TRAF2 to Inhibit PKC–NF-κB Activity in Invaded Epithelial Cells. PLoS Pathog. 2013;9: e1003409–15. doi:10.1371/journal.ppat.1003409

6. Haneda T, Winter SE, Butler BP, Wilson RP, Tükel C, Winter MG, et al. The capsule-encoding *viaB* locus reduces intestinal inflammation by a *Salmonella* pathogenicity island 1-independent mechanism. Infect Immun. 2009;77: 2932–2942. doi:10.1128/IAI.00172-09

7. Datsenko KA, Wanner BL. One-step inactivation of chromosomal genes in *Escherichia coli* K-12 using PCR products. Proc Natl Acad Sci USA. 2000;97: 6640–6645. doi:10.1073/pnas.120163297
