# Supplementary material for: A Salmonella type III effector, PipA, works in a different manner than the PipA family effectors GogA and GtgA
Source: PLoS One. 2021 Mar 18;16(3):e0248975. doi: 10.1371/journal.pone.0248975 (PMC7971870; doi:10.1371/journal.pone.0248975)
Supplement: S1 Raw images — (PDF) [file pone.0248975.s013.pdf]

Fig 3

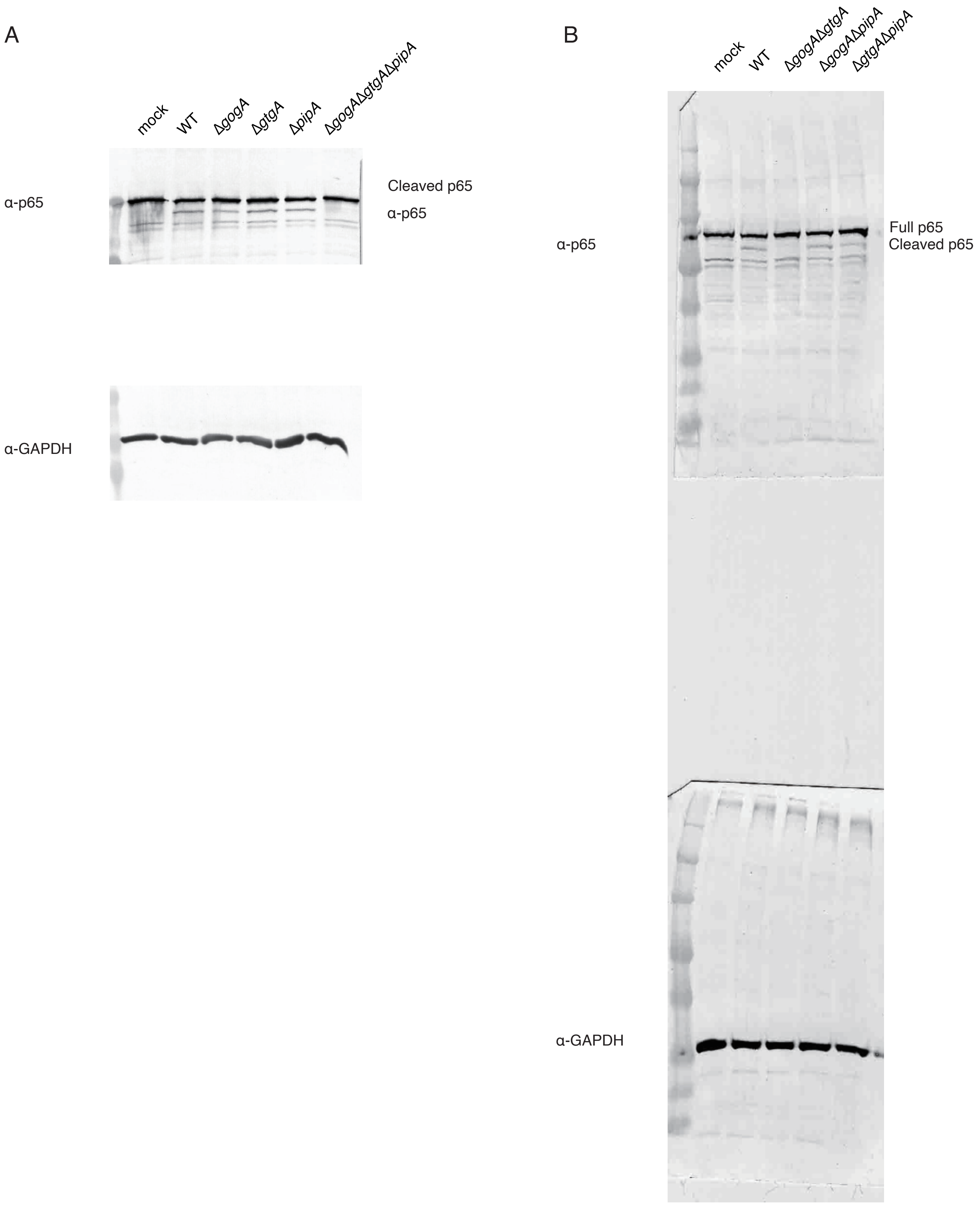
A
mock
WT
ΔgogA
ΔgtgA
ΔpipA
ΔgogAΔgtgAΔpipA
α-p65
Cleaved p65
α-p65
α-GAPDH
B
mock
WT
ΔgogAΔgtgA
ΔgogAΔpipA
ΔgtgAΔpipA
α-p65
Full p65
Cleaved p65
α-GAPDH

Fig 3

C

α-p65

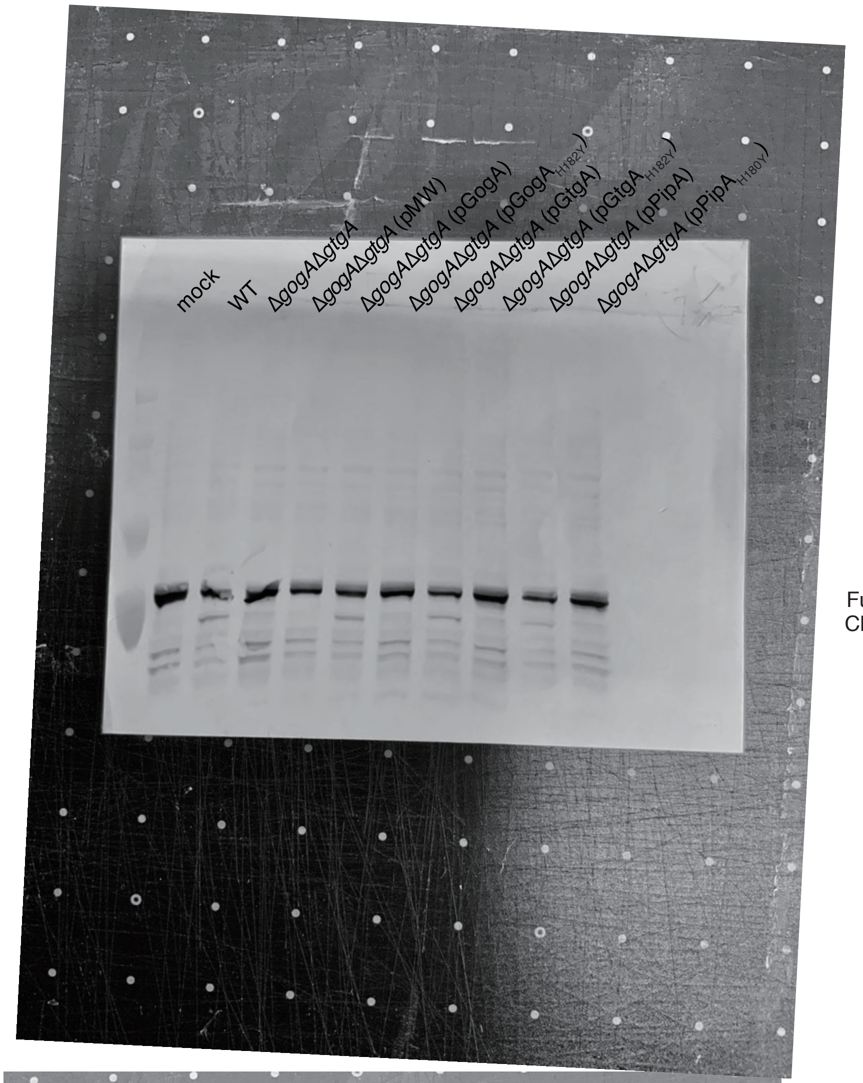

Full p65
Cleaved p65

α-GAPDH

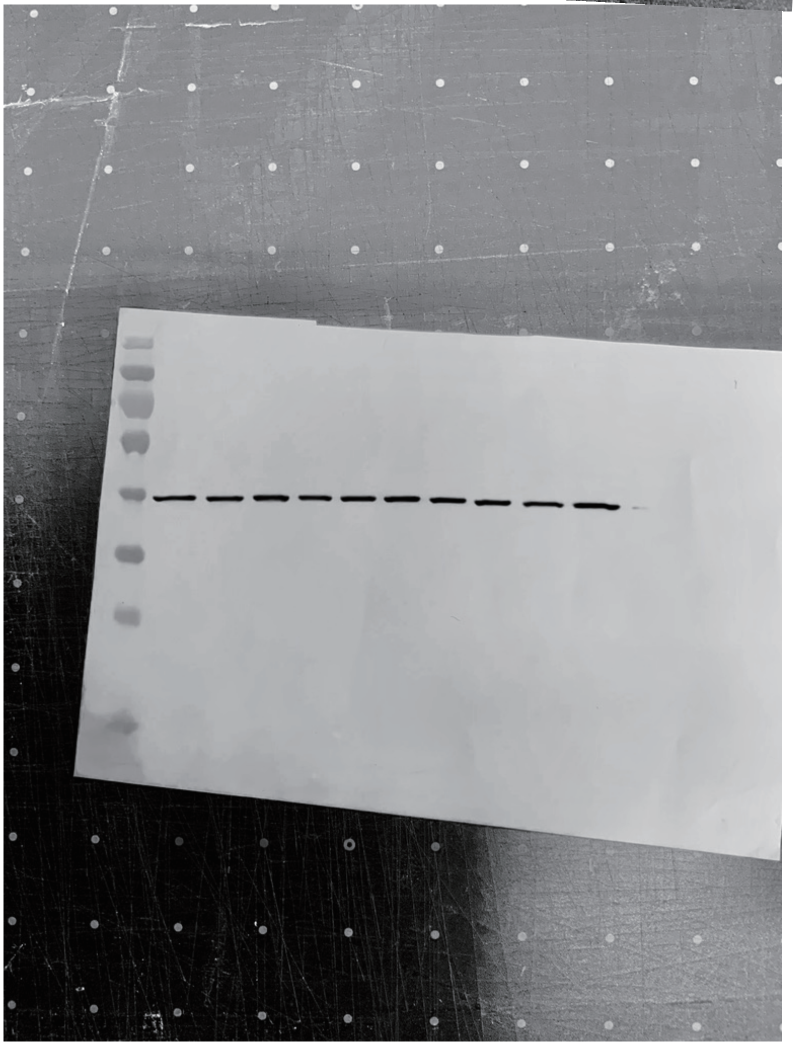

S1 Fig

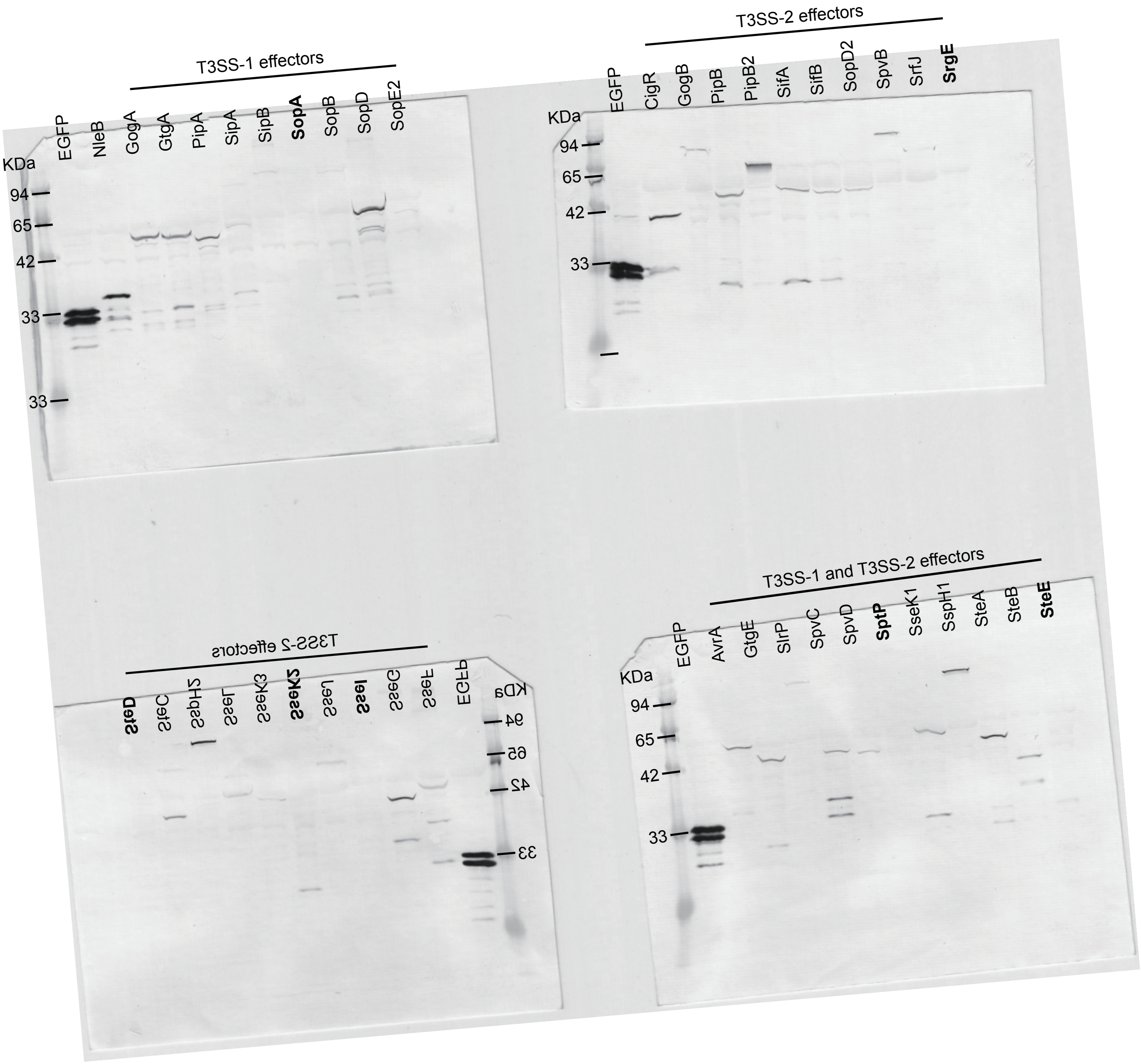
T3SS-1 effectors
KDa
EGFP
NleB
GogA
GtgA
PipA
SipA
SipB
SopA
SopB
SopD
SopE2
94
65
42
33
33
T3SS-2 effectors
KDa
EGFP
CigR
GogB
PipB
PipB2
SifA
SifB
SopD2
SpvB
SrfJ
SrgE
94
65
42
33
T3SS-2 effectors
SteD
SteC
SspH2
SseJ
SseK3
SseK2
SseL
SseI
SseG
SseF
EGFP
KDa
94
65
42
33
T3SS-1 and T3SS-2 effectors
KDa
EGFP
AvrA
GtgE
SlrP
SpvC
SpvD
SptP
SseK1
SspH1
SteA
SteB
SteE
94
65
42
33

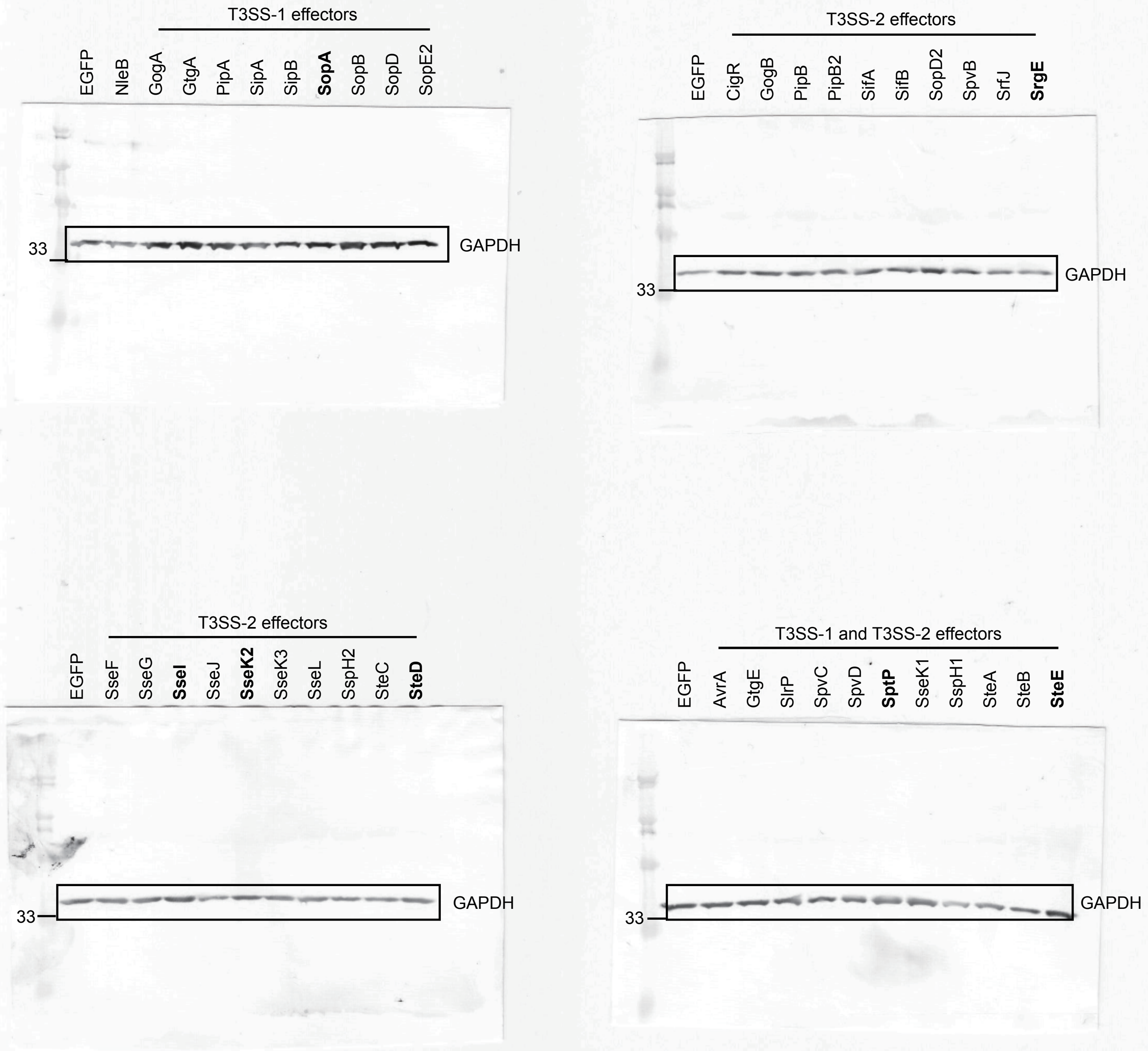
T3SS-1 effectors
EGFP NleB GogA GtgA PipA SipA SipB **SopA** SopB SopD SopE2
33
GAPDH
T3SS-2 effectors
EGFP CigR GogB PipB PipB2 SifA SifB SopD2 SpvB SrfJ **SrgE**
33
GAPDH
T3SS-2 effectors
EGFP SseF SseG **SseI** SseJ **SseK2** SseK3 SseL SspH2 SteC **SteD**
33
GAPDH
T3SS-1 and T3SS-2 effectors
EGFP AvrA GtgE SlrP SpvC SpvD **SptP** SseK1 SspH1 SteA SteB **SteE**
33
GAPDH

S2 Fig

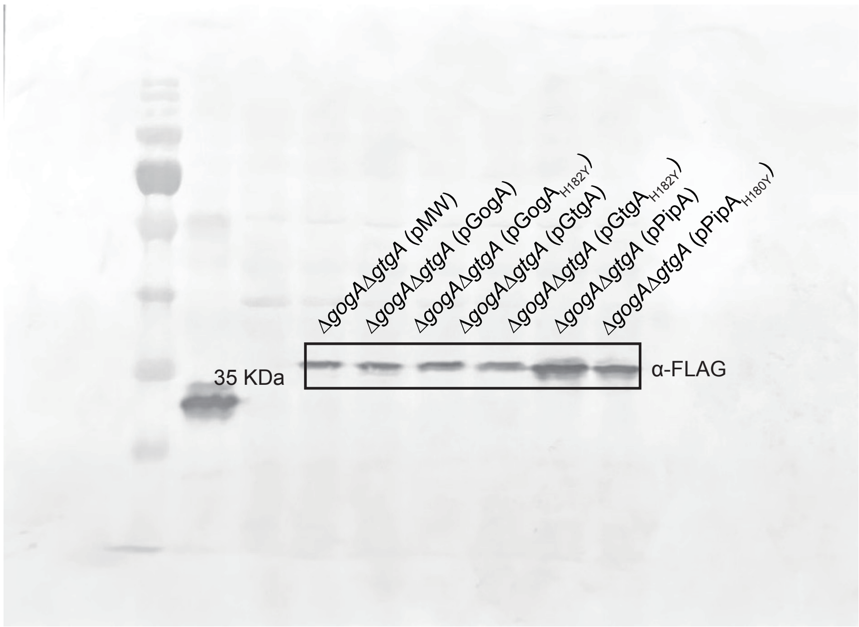

ΔgogAΔgtgA (pMW)
ΔgogAΔgtgA (pGogA)
ΔgogAΔgtgA (pGogA$_{H182Y}$)
ΔgogAΔgtgA (pGtgA)
ΔgogAΔgtgA (pGtgA$_{H182Y}$)
ΔgogAΔgtgA (pPipA)
ΔgogAΔgtgA (pPipA$_{H180Y}$)
35 KDa
α-FLAG

S3 Fig

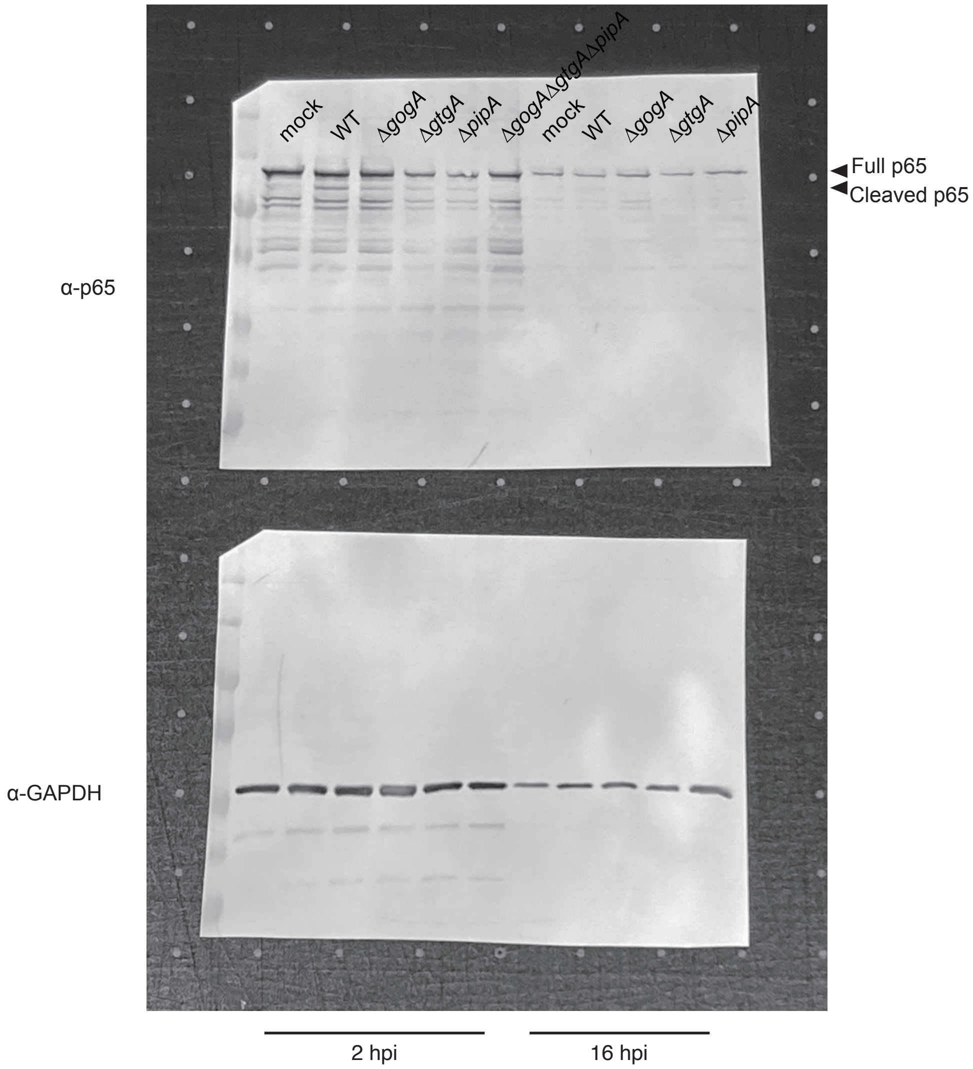

mock
WT
ΔgogA
ΔgtgA
ΔpipA
ΔgogAΔgtgAΔpipA
mock
WT
ΔgogA
ΔgtgA
ΔpipA
Full p65
Cleaved p65
α-p65
α-GAPDH
2 hpi
16 hpi

S5 Fig

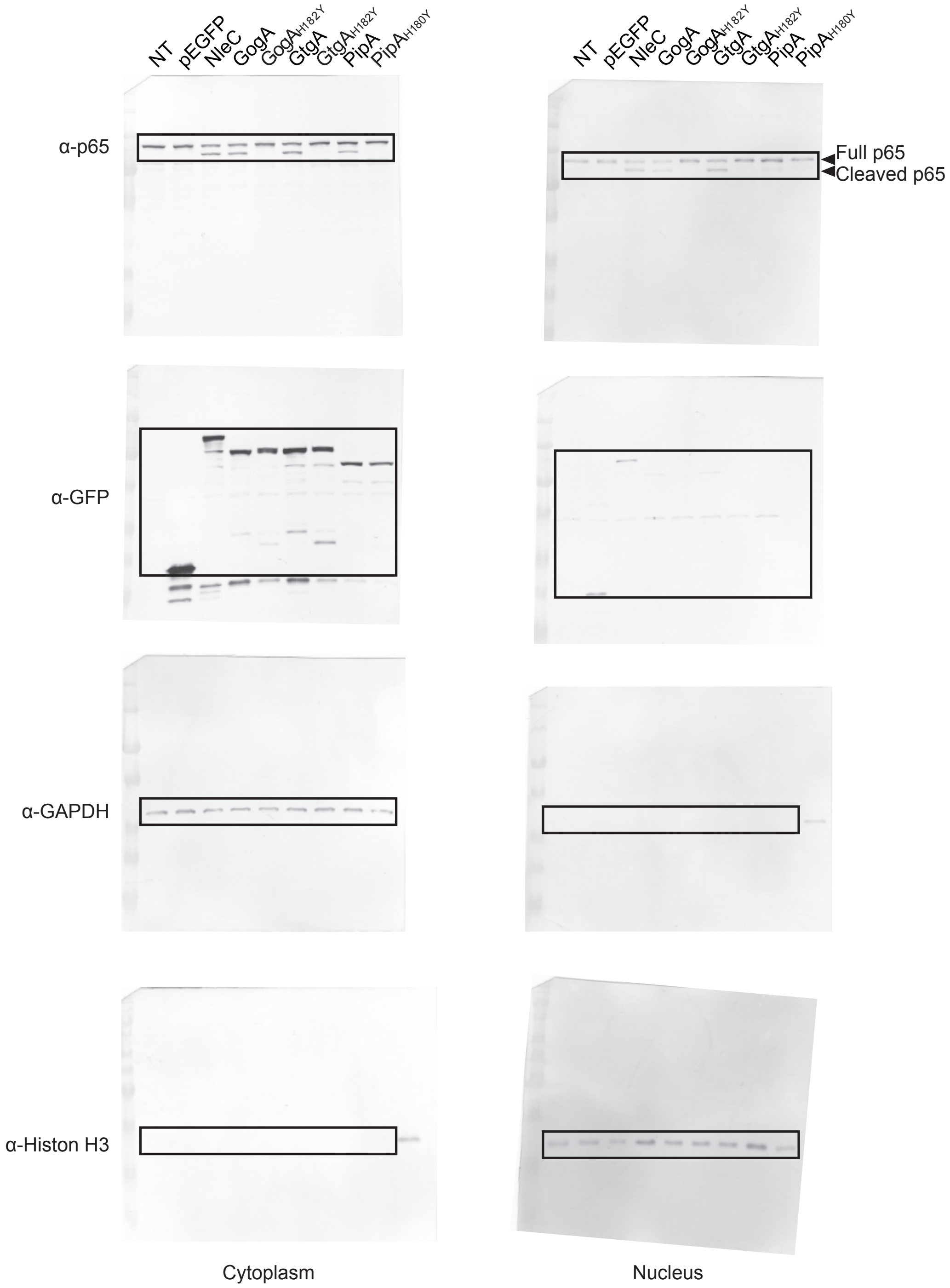
NT
pEGFP
NleC
GogA
GogA H182Y
GtgA
GtgA H182Y
PipA
PipA H180Y
α-p65
Full p65
Cleaved p65
α-GFP
α-GAPDH
α-Histon H3
Cytoplasm
Nucleus

S7 Fig

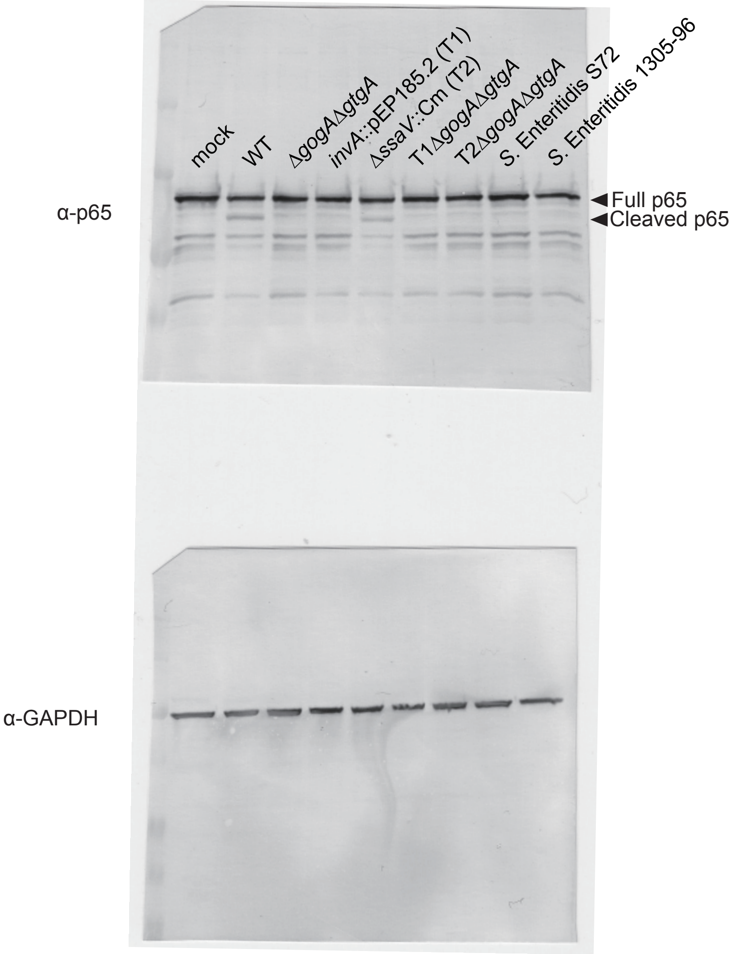

mock
WT
ΔgogAΔgtgA
invA::pEP185.2 (T1)
ΔssaV::Cm (T2)
T1ΔgogAΔgtgA
T2ΔgogAΔgtgA
S. Enteritidis S72
S. Enteritidis 1305-96
α-p65
Full p65
Cleaved p65
α-GAPDH
