# Supplementary material for: A Salmonella type III effector, PipA, works in a different manner than the PipA family effectors GogA and GtgA
Source: PLoS One. 2021 Mar 18;16(3):e0248975. doi: 10.1371/journal.pone.0248975 (PMC7971870; doi:10.1371/journal.pone.0248975)
Supplement: Attachment — Submitted filename: Response to reviewers.docx [file pone.0248975.s014.docx]

Reviewer #1: This manuscript describes the results of an overexpression screen in HeLa cells that aimed to identify Salmonella Typhimurium T3SS effector proteins which inhibit activation of an NF-κB luciferase reporter following cellular stimulation with TNFα. Haneda et al. identified seven effector proteins SseK1, SseK2, SseK3, GogA, GtgA, PipA, and SteE which inhibited NF-κB signalling when expressed ectopically. Although this is the first report to conduct such a screen for Salmonella T3SS effector proteins, several groups have already described and characterised the activities of the SseK and PipA family of effector proteins.

Using a panel of mutants deleted for gogA, gtgA, and pipA the authors conduct a more thorough analysis of which effectors inhibit NF-κB signalling in infected cells. Doing so they demonstrate that pipA might not be required for p65 cleavage or NF-κB inhibition in HeLa cells. However as only a single timepoint was analysed (Fig 3: 4 hr post invasion; Fig 4: not stated) it remains possible that PipA functions to cleave p65 at later time points post invasion. To further support this possibility, the manuscript would benefit from an analysis of NF-κB activity and/or p65 cleavage in HeLa cells infected with a gtgA gogA double mutant strain and a gtgA gogA pipA triple mutant strain in a time course assay. This is especially prudent as in Srikumar et al., 2015, pipA is maximally expressed in SPI-2-inducing conditions suggesting it might be translocated into host cells by the SPI-2 T3SS which is expressed at later time points.

Finally, the authors attempt to reproduce data from Sun et al., 2016 which demonstrates that mutant strains lacking all three members of the PipA family induce increased inflammation in the gut. Haneda et al., did not observe a difference in inflammation using several different techniques (e.g. histopathology, qPCR), however it is hard to draw conclusions from these data as the infection of CBA mice with wild-type Salmonella did not elicit inflammation as reported previously.

General comments:
- Overall the study presents new findings that appear to be of high technical standard, but the authors must provide more information in the figure legends as to the experimental N number and whether the bars represent mean and SEM? e.g. Figure 1 – what statistical test was used, what are the P values and what is the N number and is this mean and SEM? Figure 4 – at what timepoint was this analysis conducted?

RESPONSE: We appreciate the reviewer’s comment. We changed the graph type to a scatter plot overlaid on bars to indicate the N number and distribution of the samples. We also revised the figure legends as requested.

- The standard of written English through-out could be improved to aid the readers understanding of the results.

RESPONSE: We have carefully rewritten the manuscript for clarity. We also had the entire manuscript checked by a native English-speaking editor before resubmission.

- There are a number of small errors throughout, I have tried to highlight those that I have seen but please could the authors check the accuracy of their statements.

RESPONSE: We appreciate the reviewer’s comment. We have checked the entire document carefully.

- Please could the authors refer to strain genotypes in the text rather than strain ID numbers, it breaks the flow of reading to have to check back to see which strain is which (e.g. please state ∆gogA∆gtgA instead of TH1681).

RESPONSE: We have changed the strain ID numbers to strain genotypes throughout the manuscript.

- To ensure that appropriate conclusions can be drawn from the data the author should provide an α-GFP western blot to demonstrate if all the GFP-effector fusions are expressed.

Ked

RESPONSE: We added data showing that all the GFP fusion proteins were expressed in the cells (S1 Fig in the revised manuscript).

- Figure 2D and Figure 3C. Are the proteins encoded by the complementing plasmids (especially the catalytically-inactive point mutants and PipA) expressed and translocated? Please insert WB/IF analysis demonstrating if this is the case.

RESPONSE: We thank the reviewer for pointing this out. We have checked the expression of PipA or PipAH180Y on pMW118. Unfortunately, these proteins were not expressed, just as you indicated. We consider that this might be because these plasmids contain the terminator of the upstream gene (*STM14_1233* [*pipB*]) of *pipA*. We therefore reconstructed pMW-PipA and pMW-PipAH180Y, which contain not only the terminator of the *pipB* gene but also the promotor of the *pipA* gene. The expressions of these constructs in the *S*. Typhimurium ∆*gogA*∆*gtgA* strain were monitored by WB using anti-FLAG antibody (S2 Fig in the revised manuscript). The results suggest that the cleavage of NF-κB p65 by each zinc protease depends on the gene expression in the strain (Figs 2 and 3 in the revised manuscript).

Specific points
- Line 63: Inhibition of NF-κB by SseL is controversial as results of Le Negrate et al., 2008, could not be repeated by Mesquita et al., 2012. Perhaps this could be appreciated in the introduction, especially as it is a reason to conduct a non-biased screen such as that shown in Figure 1.

RESPONSE: We appreciate the reviewer’s point. We now address this in the Discussion section (lines 483-490).

- Line 68: GogB interacts with FBXO22 not HOX22.

RESPONSE: We apologize for the mistake. We changed HOX22 to FBXO22.

- Line 128: It is not clear to the reviewer which promoter drives the expression of the GogA, GtgA, and PipA complementing plasmids. Please could the authors state?

RESPONSE: We appreciate the reviewer’s point. All three genes were cloned into pMW118, which is the low copy number plasmid. This plasmid has the multiple cloning site downstream of the inducible *tac* promoter. We addressed this in the Results section in the revised manuscript (lines 316-317).

- Line 241: SteE has recently been shown by Jaslow et al., 2018 to activate STAT3 signalling. Could the authors speculate why they might see a phenotype for SteE in an overexpression screen and not in Salmonella mutants?

RESPONSE: In the revised manuscript, we showed that PipA is a T3SS-2 effector. It is reasonable that PipA does not have the phenotype in HeLa cells infected with *S*. Typhimurium after 4 hrs. T3SS-2 may not be expressed under this condition. Thus, deletions of PipA or other T3SS-2 effectors containing SteE would not show the phenotype.

- Line 251: the HExxH motif in PipA is residues 180-184.

RESPONSE: We changed 180-186 to 180-184 (line 299).

- Line 305: In Figure 4 it looks as though there is a reduction in p65 signal rather than a lack of nuclear translocation.

RESPONSE: Thank you for this comment. We agree and have changed the description accordingly (lines 356-357).

- Line 391: This sentence could be explained further. The promoter region of pipA is different, but it should be appreciated that pipA is also in an operon with a gene encoding the SPI-2 T3SS effector PipB. Perhaps also state that the N-terminal region of proteins is thought to bind to T3S apparatus/chaperones which is why the differences might suggest different secretion profiles?

RESPONSE: To confirm that PipA plays different roles than GtgA and GogA cells infected with S. Typhimurium, we first investigated whether the ∆gogA∆gtgA strains complemented with overexpressed GogA, GtgA, or PipA could interfere with the NF-κB signaling. The results showed that the ∆gogA∆gtgA strains complemented with one of these plasmids resulted in repression of NF-κB activation (Fig 2). Next, we analyzed the expressions of these three genes under conditions that induce T3SS-1 or T3SS-2. The results indicated that the pipA gene was not induced under the T3SS-1-inducing conditions (Figs 4A and 4B). These data provide an explanation for the finding that GogA and GtgA, but not PipA, inhibited NF-κB activation in HeLa cells infected with S. Typhimurium.

- Line 396: Niemann et al., does not support this statement. pipA actually looks like it is expressed in SPI-2-inducing conditions and not SPI-1-inducing conditions. Check Srikumar et al., 2015/SalCom database.

RESPONSE: We appreciate the reviewer’s comment. As mentioned above, we confirmed that the *pipA* gene is induced under the T3SS-2-inducible condition, but not under the conditions which induce T3SS-1. This result is consistent with the previous report (Srikumar et al., 2015).

- Line 404-406: As presently written this sentence could be interrupted to mean that STMUK_2648 is a homolog of GogA, GtgA, and PipA which is not the case. STMUK_2648 is a homolog of NleC.

RESPONSE: We added additional text to prevent misreading and clarify that STMUK_2648 is a homolog of NleC (lines 512-513).

Reviewer #3: The manuscript by Takemura et al. describes the involvement of three related effectors of S. Typhimurium (STm) to reduction of NF-kB mediated inflammatory response in HeLa cells and mouse infection model. Although the inhibitory role of the three effectors has been published in 2016, the involvement of each effector remained unsolved. This study presents the differences between one of effector, PipA from the other two homologs, GogA and GtgA, in protease activity and inhibitory effect on NF-kB signaling. The manuscript also presented the results of mouse infection studies without any contribution of the three effectors in STm infection, which seem to be inconsistent with the previous report by Sun et al. Experimental to clarify the roles of three effectors were well designed, though interpretations of results are somewhat unconvincing. First of all, I feel concern on the expression of effector genes in STm. If the expression of pipA gene was lower than gogA and gtgA, most of results can be explained. Further, the activity of p65 cleavage also can be affected by the differences in efficiency of production in cells.

Concerning the experiment of Fig2D, why induction of pipA can not rescue the mutation? While ectopic expression clearly reduced the NF-kB response as shown in Fig1. One of explanation could be that the expression of pipA from the plasmid is poor.

RESPONSE: We appreciate the reviewer’s comment. We checked the expression of PipA on pMW118. Unfortunately, PipA was not expressed. We then constructed additional plasmids, pMW-GogA-FLAG, pMW-GtgA-FLAG and pMW-PipA-FLAG, in order to monitor the expression of these proteins as FLAG-fusion proteins. As shown in S2 Fig in the revised manuscript, the GogA-, GtgA-, and PipA-FLAG fusion proteins were all expressed in the ∆*gogA*∆*gtgA* strain.

Which of T3SS-1 or T3SS-2 is the main secretion machinery of PipA? If T3SS-2 is the main machinery for PipA, results of HeLa-infection experiments can be explained by timing of usage.

RESPONSE: We checked GogA, GtgA and PipA expression under the T3SS-1- and T3SS-2-inducing conditions in vitro (Figs 4A and 4B in the revised manuscript). The results suggested that GogA and GtgA could be both T3SS-1 and T3SS-2 effectors, but PipA could be a T3SS-2 effector but not a T3SS-1 effector. Thus, it seems reasonable to conclude that PipA cannot cleave p65 in HeLa cells infected with S. Typhimurium in the early stage of infection.

In cells infected with wild type STm, though the cleaved p65 appeared, full size of p65 remained at the comparable level to cell infected with triple mutant strain or pipA mutants as shown in Fig3. Why were the NF-kB activities of WT and gogA/gtgA mutants infected cells much lower than triple or pipA mutants infected cells?

RESPONSE: We apologize for any confusion. Under our experimental conditions, the *S*. Typhimurium wild-type strain entered into only <2% of inoculation. This indicates that because the intact (full) p65 is detected in almost of the cells by immunoblotting, the levels of full p65 in the cells infected with every strain, even the wild-type and triple mutant, may not be comparable. However, cleaved p65 could be clearly detected in the cells that were infected with S. Typhimurium expressing any one of GogA, GtgA, and PipA. In the reporter assay, pGL4.32, which has the response element that drives transcription of the luciferase reporter gene, was transfected in HeLa cells. That is, only cells in which the NF-κB pathway was activated was detectable. These data indicate that GogA, GtgA, and PipA can cleave p65, and that the expression of at least one of these effector genes is required for blocking of NF-κB signaling.

Concerning the mouse infection experiments, infection efficiency in this study seems lower than the experiments by Sun et al. In spite of higher number of bacteria (10^9 cfu) were orally infected numbers of bacteria in spleen were lower by more than one order of magnitude, as compared to Sun's experiments, in which 10^8 were used for infection. Is it likely that inflammatory response was induced at low level because of lower infection efficiency in these experiments? This could be a reason of discrepancy of the results.

RESPONSE: We appreciate the reviewer’s comment. The different susceptibility of mice to infection of *S*. Typhimurium between this study and Sun’s experiment may have been related to the different *S*. Typhimurium wild-type strains used, ATCC14028 and SL1344. The difference might also be explained by colonization resistance. Rivera-Chaves et al. also reported bacterial numbers (10^7 CFU/g at day 4 and 10^8 CFU/g at day 7) in feces from CBA mice infected with IR715, which is a spontaneously nalidixic acid-resistant strain of S. Typhimurium 14028, that were higher than those in our experiments (Cell Host Microbe, 2016). In the revised manuscript, we added a brief treatment of the discrepancy in the Discussion section (lines 536-541).

Reviewer #4: Takemura et al. identified 7 Salmonella enterica serovar Typhimurium effectors that block NFkB signaling in HeLa cells. With the exception of one effector, their effect on NFkB signaling was previously known. The authors analyzed the effect of three effectors on NFkB signaling in detail: PipA, GtgA and GogA. Interestingly, they found that the GtgA and GogA, but not PipA block NFkB activation in HeLa cells. GogA and GtgA readily cleaved NFkB p65, while PipA cleaved NFkB p65 to a lesser extent. The authors analyzed virulence of a gogA gtgA pipA deficient strain in CBA mice. Contrary to previously reported C57BL/6 Nramp functional mice, they did not see differences compared to wild type infected mice (CFU and inflammation).

The authors performed their experiments rigorously and included all relevant controls, including Salmonella mutants encoding catalytically inactive effector versions. Importantly, the authors did not overstate their results and compared and contrasted them to what is already known in the literature. This is particularly important, as much is known about these effectors already. Compared to published studies, the authors report a few differences.

The authors should address the following points:

1) It is not clear in all graphs, to which comparisons “NS” apply. Does a line over three bars mean that there is no significance between bar 1 and bar 3 or that there are no significant differences between all three bars? If all comparisons other than the ones marked are not significant, please add this to the figure legend. Some comparisons seem like there should be statistical differences. Did the authors elect to not show certain comparisons to keep the clarity of the figures?

RESPONSE: We agree with the reviewer and have incorporated this suggestion throughout the revised manuscript.

2) The only statistical method for cell-based assays is student’s T-test, which was used for pairwise individual comparisons. I am not a statistician, but ANOVA should be used in e.g. Fig. 1 where multiple groups are compared. The authors should make sure appropriate statistical tests are used.

RESPONSE: We re-analyzed our data in Figure 1 using an unpaired ANOVA, as suggested.

3) The figure legends should indicate which statistical test was used and what stars mean. In Fig. 1 it is not clear what the star indicates (statistically different compared to what?).

RESPONSE: We revised the figure legends to clarify what the stars mean throughout the manuscript.

4) The authors cautiously analyzed their in vivo data, as they saw no significant differences between groups. However, there are trends that should be mentioned. The effector mutant has a 1 log higher colonization in the cecum. Even though there is no significance, this also corresponds to moderate inflammation in these mice compared to wild type infected mice.

RESPONSE: We appreciate the reviewer’s comment. As suggested, along with the higher colonization in the ceca of mice infected with ∆gogA∆gtgA compared to the ceca of those infected with the wild-type at 7 days, moderate inflammation tended to be induced. This is now mentioned in the revised manuscript (lines 420-422).

5) Line 323: There is a faint band for cleaved p65 in the PipA lane. Later, the authors acknowledge this, so it should also be mentioned here.

RESPONSE: We appreciate the reviewer’s comment. In the revised manuscript, we make the claim that PipA does not work under a T3SS-1-inducing condition rather than the claim that PipA has less enzymatic activity compared to GogA or GtgA. Thus, we moved Fig 5 in the original manuscript to the Supplemental figures (S4 Fig). We revised the text to explain that GogA, GtgA, and PipA have metalloprotease activity consistent with previous studies (lines 362-364).
