# Supplementary material for: A Salmonella type III effector, PipA, works in a different manner than the PipA family effectors GogA and GtgA
Source: PLoS One. 2021 Mar 18;16(3):e0248975. doi: 10.1371/journal.pone.0248975 (PMC7971870; doi:10.1371/journal.pone.0248975)
Supplement: Attachment — Submitted filename: Response to Reviewers.docx [file pone.0248975.s015.docx]

Reviewer #1: Please see enclosed the review of “A Salmonella Type III effector, PipA, works in a different manner than the PipA family effectors GogA and GtgA.” This well-written manuscript demonstrates that purified GogA, GtgA, and PipA reduce NF-kB signaling in tissue-cultured epithelial cells due to cleavage of NF-kB p65 subunit but only GogA and GtgA are required for the cleavage in these cells, likely due to secretion through the T3SS-1. While the PipA protein can cleave NF-kB p65, it is not active during infection with S. Typhimurium, likely due to secretion through the T3SS-2. The roles of PipA, GogA, and GtgA appear to be non-redundant during murine infection. The methodology lack details and/or clarity and there are numerous discrepancies noted below that require clarification. Since in vitro culture conditions can have a great impact on expression of T3SS-1 and T3SS-2 genes and resulting impact on host-pathogen interactions, a complete description of the methodology used for the reported work will facilitate understanding of the presented work as well as placement into context of prior work on the same genes and processes.

Major points:

For all figures: The description of each figure panel in the legend would improve the readers’ understanding of the figures as stand-alone data. Furthermore, statement of the number of technical and biological repeats each data point represents would allow for improved assessment of experimental rigor.

RESPONSE: We appreciate the reviewer’s comment. We revised all figure legends for clarity, with the exception of Fig 6.

The authors state (Lines 263, 273, 323-325, 342, 493) that PipA does not dampen NF-kB production, but this statement is not supported by the data presented in Figures 2D and 3C, which demonstrate that PipA can compensate for loss-of-function of both GtgA and GogA. Please clarify.

RESPONSE: We thank the reviewer for pointing this out. As shown in Fig 4A, PipA is not expressed in the early stage of infection of *S*. Typhimurium. This is the reason why the ∆*gogA*∆*gtgA* strain does not suppress the NF-κB activation and cleave p65 in HeLa cells infected with *S*. Typhimurium, although this strain has a *pipA* gene (Figs 2C and 3C). However, the ∆*gogA*∆*gtgA* (pPipA) strain, which overexpresses PipA from the plasmid, dampens the NF-κB activation. These facts suggest that the PipA protein would potentially dampen the NF-κB activation by cleaving p65. We now address this in the Discussion section (lines 528–531).

The implication of PipA as a T3SS-2 effector comes from secretion assays (Figure 4C) as well as intracellular replication and cytotoxicity assays. Furthermore, prior work suggests that PipA is expressed in T3SS-2-inducing conditions (reference 45). However, the T1 PipA mutant behaves as the T1 mutant, not as a T1T2 mutant, suggesting no role for PipA in T3SS-2 function in RAW cells. Establishing the role of PipA in a ∆T3SS-2 mutant would more closely link PipA to the T3SS-2 function through genetic means. Furthermore, by linking PipA with T3SS-2, evaluation of PipA activity in HeLa cells at 4hpi is too early, as the T3SS-2 is not active at that time. Evaluation of PipA activity on NF-kB cleavage at a time when T3SS-2 is expressed would improve the clarity of the argument regarding PipA and T3SS-2 function. Furthermore, amendment of the discussion regarding the function of PipA in HeLa cells during early infection would clarify the discrepancies between overexpression and infection conditions in these cells.

RESPONSE: We appreciate the reviewer’s comments. We added data on the NF-κB activity of HeLa cells infected with the wild-type or the ∆*gogA*∆*gtgA*∆*pipA*∆*sseK1*∆*sseK2*∆*sseK3*∆*steE* strain after 20 hrs of infection (Fig 2A). These data show that effectors other than the five identified effectors might attenuate the NF-κB signaling pathway at 20 hrs after infection. Previous studies have shown that other T3SS-2 effectors, i.e., AvrA, GogB, SpvD, SseL, SspH1 or SteA, inhibit the NF-κB activation (references 8–12, 47). These effectors inhibit the NF-κB signaling pathway in a way that is different from cleaving p65. Interestingly, in addition to PipA, most of these other effectors also have no effect on intracellular survival and induction of cell death in RAW cells infected with *S*. Typhimurium. These data might indicate that these T3SS-2 effectors have overlapping functions in the *S*. Typhimurium-infected cells. A mutant in which all of these effectors are deleted will be needed to understand the role of the inhibition of the NF-κB signaling in the late stage of *Salmonella* infection. We revised the Discussion section to incorporate these points (lines 515­­–524).

We also added the immunoblotting data related to the detection of cleaved p65 in HeLa cells after 16 hrs of infection (Fig S3). However, we did not observe cleaved p65 in HeLa cells infected with the wild-type or any of the mutants used in this experiment. This data may support the hypothesis that PipA digests another target in the late stage of *Salmonella* infection (lines 561–562).

Lines 309-313: The results presented in Figure 2C demonstrate a role for GtgA alone in NF-kB suppression, but GogA has no effect on its own, although there appears to be an additive effect of GogA and GtgA. These results merit discussion as to potential redundancy/lack thereof of these two effector proteins.

RESPONSE: We appreciate the reviewer’s comment. The level of NF-κB activity of HeLa cells infected with the ∆*gogA* strain is significantly higher than that of the wild type strain, but that of HeLa cells infected with the ∆*gtgA* strain is not. However, the NF-κB activity of the cells infected with the ∆*gtgA* or ∆*gogA* strain is lower than that of cells infected with the ∆*gogA*∆*gtgA* or ∆*gogA*∆*gtgA*∆*pipA* strain, both of which are incapable of p65 cleavage. We also showed that not only the ∆*gtgA* and ∆*pipA* strains but also the ∆*gogA* strain cleaves NF-κB p65 (Fig 3A). This indicates that the level of the NF-κB activity of the cells infected with the ∆*gtgA* strain is sufficient to show cleavage of p65. We now address this in the Discussion section (lines 502–507).

Minor points:

Lines 102: The authors mention a “slight modification…” of prior scoring system. Please describe the modification.

RESPONSE: We added a description of our modification in the Materials and Methods section as requested (lines 103–107).

Line 138: pFLAG-CTC is not listed in S2 table.

RESPONSE: We added pFLAG-CTC and its derivatives in Table S2.

Lines 202-203: Please describe culture conditions for stimulation of T3SS-1 and T3SS-2 with respect to osmolarity, oxygenation, and media composition. LPM acronym should be spelled out.

RESPONSE: We added the culture conditions in detail in the Materials and Methods section as requested (lines 208–210).

Lines 254-5: The details regarding TNF-a stimulation and duration conflict with those stated in the methods (lines 164-5). Please clarify.

RESPONSE: We corrected the duration of treatment with TNF-α in the Materials and Methods section (line 170).

Lines 268-9: The timing of NF-kB measurement relative to S. Typhimurium infection conflict with that in line 171. Furthermore, please describe the culture conditions for induction of T3SS-1 (line 170).

RESPONSE: We revised the timing of measurement of the NF-κB activity (line 176). We also added the culture conditions for induction of T3SS-1 (line 175) .

Lines 316-318 and Figure 2D: The methodology for induction of gene expression is not provided in the text. Please correct.

RESPONSE: We added the methodology for induction of gene expression in the Materials and Methods section and the legend of Fig 2D (lines 208–210 and line 284).

Figure 4A and 4B: Y-axis indicates normalization of target to gapdh, which is not a bacterial gene. Please correct.

RESPONSE: We changed *gapdh* to *gyrA* on the Y-axis in Figs 4A and 4B.

Lines 378-381: Please indicate how induction of gogA/gtgA and pipA were determined. It is unclear from the text and figure legend what comparators were used to establish induction vs. no change in gene expression in a given condition.

RESPONSE: We indicated how induction of these genes was determined (lines 393–395).

Lines 412, 416, and others. Mice have one cecum, therefore it is correct to refer to mouse cecum, rather than mouse ceca. Please correct.

RESPONSE: We corrected *mouse ceca* to *mouse cecum*.

Line 421: the text mentions colonization in the cecum, but methods and figure axes indicate colonization in colon contents. Please clarify.

RESPONSE: We corrected *cecum* to *colon* *contents* (line 442).

Figure 6: Please indicate whether the CI calculation indicates mutant/WT or vice versa to improve the readers’ understanding of the presented data.

RESPONSE: We changed each graph title in Fig 6 from *WT vs. mutant* to *mutant vs. WT*. And we also added mutant/WT to the Y-axis.

supporting information: The legends for Figures S2-5 do not match the figures. Similarly, there are no figure legends for Figures S7 or S8. Please correct.

RESPONSE: We revised the legends for Figs S2–5 (Fig S2 and S4–6 in the revised supporting information) and added the legends for Fig S7 and S8 (Figs S8 and S9 in the revised supporting information).

Reviewer #2: The manuscript by Takemura et al. identifies seven S. Typhimurium effectors that when overexpressed in HeLa cells, inhibit NF-kB signaling. Although effectors of the SseK and PipA family have previously been reported to target NF-kB activity, Takemura et al. here report for the first time that the effector SteE may also contribute to inhibiting NF-kB signaling. Using a series of Salmonella mutants and complemented strains the authors show that effector mediated inhibition of NF-kB by the PipA family of zinc metalloproteases GogA, GtgA and PipA cleaves the p69 subunit in a manner dependent on the induction of two Salmonella T3SS (T3SS-1 and T3SS-2 for GogA and GtgA and T3SS-2 for PipA).

The authors also sought to recapitulate a previously reported enhanced colitis induced by the gogA gtgA pipA S. Typhimurium mutant in an Slc11a1+/+ animal genetic background but were unsuccessful likely due to increased colonization resistance by the microbiota of their animals. Takemura et al., do however find that PipA protease activity does enhance systemic dissemination of S. Typhimurium in accordance with a previous observation by Knodler et al., (2002) that PipA is required for virulence in BALB/c mice.

The experiments are well designed and rigorously performed and statistical methods applied are in accordance with standards in the field. Clarity in the manuscript could be improved by addressing the following specific points:

1. Line 325: More specific language should be used to describe the cells referenced in this line. It is not clear if the authors mean the gogA gtgA Salmonella mutant cells or HeLa cells.

RESPONCSE: We appreciate the reviewer’s comment. We changed the cells to the ∆*gogA*∆*gtgA* strain to clarify our meaning in this sentence (line 356).

2. Line 396: Here the T1 PipA strain is first introduced and it’s genotype (invA::pEP185.1 pipA::Km) should be included (as it is in line 443) so as to not confuse it with a strain that overexpresses PipA.

RESPONSE: We changed *T1 PipA* to *invA::pEP185.1 pipA::Km* (line 416).

3. Line 442: The complete strain name (C57BL/6) of the black 6 animal should be used instead of the term B6.

RESPONSE: We changed *B6* to *C57BL/6* (line 466).

4. The rational for using the invA mutant background strains for the animal infections for Figure 6 should be briefly explained in the text.

RESPONSE: We added the reason why the strains were used in the mouse experiment (lines 462–465).

5. Line 479: The authors reference the spiB mutant yet Figure S7 indicates that the ssaV mutant was used. Please revise the text or figure to accurately reflect which strain was used in this experiment.

RESPONSE: We revised the text to indicate that the *ssaV*::Cm mutant was used (line 509).

6. The figure legends for S7 and S8 are missing from the text. Please add figure legends for these supplemental figures.

RESPONSE: We added figure legends for Figs. S7 and S8 (Figs S8 and S9 in the revised supporting information).
