# Supplementary material for: A Salmonella type III effector, PipA, works in a different manner than the PipA family effectors GogA and GtgA
Source: PLoS One. 2021 Mar 18;16(3):e0248975. doi: 10.1371/journal.pone.0248975 (PMC7971870; doi:10.1371/journal.pone.0248975)
Supplement: Attachment — Submitted filename: Response to Reviewers.docx [file pone.0248975.s016.docx]

Reviewer #1: The revised manuscript entitled “A Salmonella type III effector, PipA, works in a different manner than the PipA family effectors GogA and GtgA” has addressed most reviewer comments and is improved in clarity. The added data support the authors’ hypothesis that PipA, while capable of cleavage of p65, is unlikely to perform this function in HeLa cells during early infection. However, there are a few points that require clarification, as described below:

Minor points:

1. Plasmids used for expression of EGFP-fusion proteins in Figures 1 and S1 are incompletely described. Tables S2 and S3 describe plasmids containing GtgA, GogA, and PipA only. Please indicate primers/plasmids used to generate data for Figures 1 and S1, or reference study producing the constructs.

Response: We added primers and plasmids as requested.

2. Lines 169-70 and 261-2 and 32 in supporting information (Fig S4 legend) conflict as to TNF-a stimulation.

Response: We corrected “20 ng/ml” to “10 ng/ml” in line 169.

3. Data regarding bacterial colonization in the cecum are not presented in Figure 5A (line 444). Please correct.

Response: We corrected “cecum” to “colon contents” in line 444.

4. Lines 508-511 provide new experimental results in support of data in Fig 4. I recommend moving these data to results section in support of T3SS-1 secretion as important for early p65 cleavage.

Response: We appreciate the reviewer’s comment. However, S8 Fig. should be left as is if possible. In Fig 4, it shows that PipA is a T3SS-2 effector rather than that GogA and GtgA are T3SS-1 effectors. On the other hand, S8 Fig shows not only that GogA and GtgA cleave p65 in T3SS-1 dependent manner, but also that the inactivation of NF-κB by the GogA and GtgA are the *S*. Typhimurium-specific phenotype in *Salmonella*. It has been published that GogA and GtgA function as T3SS-1 effectors and the genes encoding GogA and GtgA are located on prophages specifically integrated in the genome of *S.* Typhimurium. Thus, the data on S8 Fig. support Figs 2 and 3, and the previous reports.

5. Lines 526-531. There is an apparent discrepancy between sentences in 526-7 and 530-1. I suggest clarification in lines 530-1 that the PipA protein can cleave p65 but does not appear to do so at the times measured in HeLa cells during S. Typhimurium infection.

Response: We revised the sentence as suggested.
